# RIPK1 activation mediates neuroinflammation and disease progression in multiple sclerosis

**DOI:** 10.1016/j.celrep.2021.109112

**Published:** 2021-05-11

**Authors:** Matija Zelic, Fabrizio Pontarelli, Lisa Woodworth, Cheng Zhu, Amy Mahan, Yi Ren, Michael LaMorte, Ross Gruber, Aislinn Keane, Pequita Loring, Lilu Guo, Tai-he Xia, Boyao Zhang, Pontus Orning, Egil Lien, Alexei Degterev, Timothy Hammond, Dimitry Ofengeim

**Affiliations:** 1 Sanofi, Neurological Diseases, 49 New York Ave., Framingham, MA 01701, USA; 2 Sanofi, Translational Sciences, 49 New York Ave., Framingham, MA 01701, USA; 3 Department of Cell, Molecular & Developmental Biology, Tufts University School of Medicine, Boston, MA 02111, USA; 4 Program in Innate Immunity, Division of Infectious Diseases and Immunology, Department of Medicine, University of Massachusetts Medical School, Worcester, MA 01605, USA; 5 Centre of Molecular Inflammation Research, Department of Clinical and Molecular Medicine, Norwegian University of Science and Technology, Trondheim, Norway; 6 Lead contact

## Abstract

Receptor interacting protein kinase 1 (RIPK1) mediates cell death and inflammatory signaling and is increased in multiple sclerosis (MS) brain samples. Here, we investigate the role of glial RIPK1 kinase activity in mediating MS pathogenesis. We demonstrate RIPK1 levels correlate with MS disease progression. We find microglia are susceptible to RIPK1-mediated cell death and identify an inflammatory gene signature that may contribute to the neuroinflammatory milieu in MS patients. We uncover a distinct role for RIPK1 in astrocytes in regulating inflammatory signaling in the absence of cell death and confirm RIPK1-kinase-dependent regulation in human glia. Using a murine MS model, we show RIPK1 inhibition attenuates disease progression and suppresses deleterious signaling in astrocytes and microglia. Our results suggest RIPK1 kinase activation in microglia and astrocytes induces a detrimental neuroinflammatory program that contributes to the neurodegenerative environment in progressive MS.

## In brief

Zelic et al. characterize RIPK1-kinase-dependent regulation of inflammation and cell death in microglia and cell-death-independent inflammatory signaling in astrocytes. They demonstrate detrimental non-cell-autonomous consequences on oligodendrocytes and use animal models and human tissue to establish the involvement of RIPK1 in progressive forms of multiple sclerosis.

## Graphical abstract

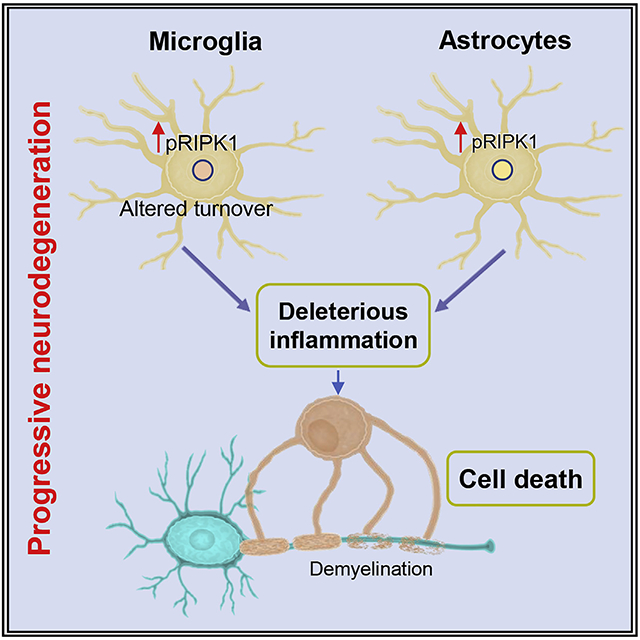

## INTRODUCTION

Multiple sclerosis (MS) is a chronic autoimmune disease of the central nervous system (CNS) characterized by neuroinflammation, demyelination, and axonal degeneration. Although most patients are initially diagnosed with relapsing/remitting MS(RRMS), many patients have worsening symptoms over time and gradual progression to secondary progressive MS (SPMS) (Inojosa et al., 2021). A subset of patients present with primary progressive MS (PPMS) at diagnosis. Although the time courses of SPMS and PPMS are markedly different, the underlying pathologies are similar (Lassmann, 2019). Current therapies are efficacious in treating symptoms associated with RRMS, but therapeutic options for progressive disease remain limited (Ciotti and Cross, 2018; Lassmann, 2017). A key aspect thought to regulate MS progression is the mechanism by which the CNS responds to peripheral immune infiltration. Microglia are the most abundant immune cells in active MS lesions (Kuhlmann et al., 2017); their altered function and ability to interact with other CNS-resident cells, like astrocytes, is a critical determinant of the CNS milieu in disease. Microglia rapidly proliferate in response to CNS insults, and their turnover may be a regulator of CNS homeostasis (Fuger et al., 2017; Hagan et al., 2020; Madore et al., 2020). Astrocytes can also contribute to pathology in MS and other neurodegenerative diseases (Liddelow et al., 2017; Ponath et al., 2018a; Wheeler and Quintana, 2019), but the interplay of these two cell types in the context of MS has not been fully explored.

Receptor interacting protein kinase 1 (RIPK1) is a serine/threonine kinase that regulates homeostasis at cellular and tissue levels by integrating inflammatory and cell death signaling (Dillon et al., 2014; Yuan et al., 2019). In the CNS, RIPK1 is expressed in all major cell types (Ofengeim et al., 2015; Zhang et al., 2014). RIPK1 kinase activity has been implicated in the pathology associated with CNS diseases, including Alzheimer’s disease (Ofengeim et al., 2017), amyotrophic lateral sclerosis (ALS) (Ito et al., 2016; Xu et al., 2018), Niemann-Pick disease type C (Cougnoux et al., 2018), and MS (Harris et al., 2019; Ofengeim et al., 2015; Yoshikawa et al., 2018; Zhang et al., 2019). The kinase activity of RIPK1 regulates both caspase-independent necroptotic death via RIPK3 and mixed lineage kinase domain-like (MLKL) and RIPK1-kinase-dependent apoptosis (RDA) via caspase-8, and emerging evidence suggests RIPK1 can also regulate proinflammatory cytokine and chemokine production (Najjar et al., 2016; Zhu et al., 2018). Thus, in the context of neurodegeneration, RIPK1 may be a cell death regulator that can also cause a detrimental neuroinflammatory environment. However, it is unclear whether differential signaling exists downstream of distinct RIPK1-activating stimuli and what the cellular contribution to inflammation and cell death is in the context of neurodegeneration and MS disease progression. Our data suggest RIPK1 can be activated in both microglia and astrocytes in MS. We show that unlike in microglia, in which RIPK1 activity mediates inflammation and cell death, astrocytes have lower MLKL expression and do not undergo necroptosis. However, RIPK1 activation mediates a strong inflammatory response in astrocytes, suggesting RIPK1 can propagate deleterious inflammation. Importantly, astrocyte resistance to RIPK1-kinase-mediated cell death allows clear uncoupling of inflammatory signaling from cell-death-associated inflammation. We used an RNA sequencing (RNA-seq) approach to better understand RIPK1-kinase-dependent regulation. By interrogating multiple CNS cell types, we derive a core RIPK1-kinase-dependent transcriptional signature and demonstrate its translational relevance to inflammatory and MS pathogenesis.

Our data indicate RIPK1 expression and activity are increased in progressive MS. To mimic the CNS milieu, we used a mixed culture system containing microglia and astrocytes and demonstrated that non-cell-autonomous signaling results in loss of oligodendrocyte precursor cells (OPCs) and reduced myelination in a RIPK1-kinase-dependent manner. These data suggest RIPK1 kinase activation creates an inflammatory CNS environment that results in oligodendrocyte dysfunction and accelerates MS progression. Similar to previous reports (Ofengeim et al., 2015; Yoshikawa et al., 2018), we show RIPK1 kinase inhibition attenuates disease in the C57BL/6 experimental autoimmune encephalomyelitis (EAE) model of MS. Therapeutic administration of a RIPK1 inhibitor attenuates disease phenotype in a dose-dependent manner. Furthermore, we identify a RIPK1-kinase-dependent gene signature in both microglia and astrocytes during EAE. Finally, we use the non-obese diabetic EAE (NOD-EAE) mouse model to examine the role of RIPK1 in the progression of clinical symptoms. We show RIPK1 expression is increased and demonstrate that RIPK1 kinase inhibition slows disease progression.

## RESULTS

### RIPK1 signaling is upregulated in progressive MS

**Figure 1.**
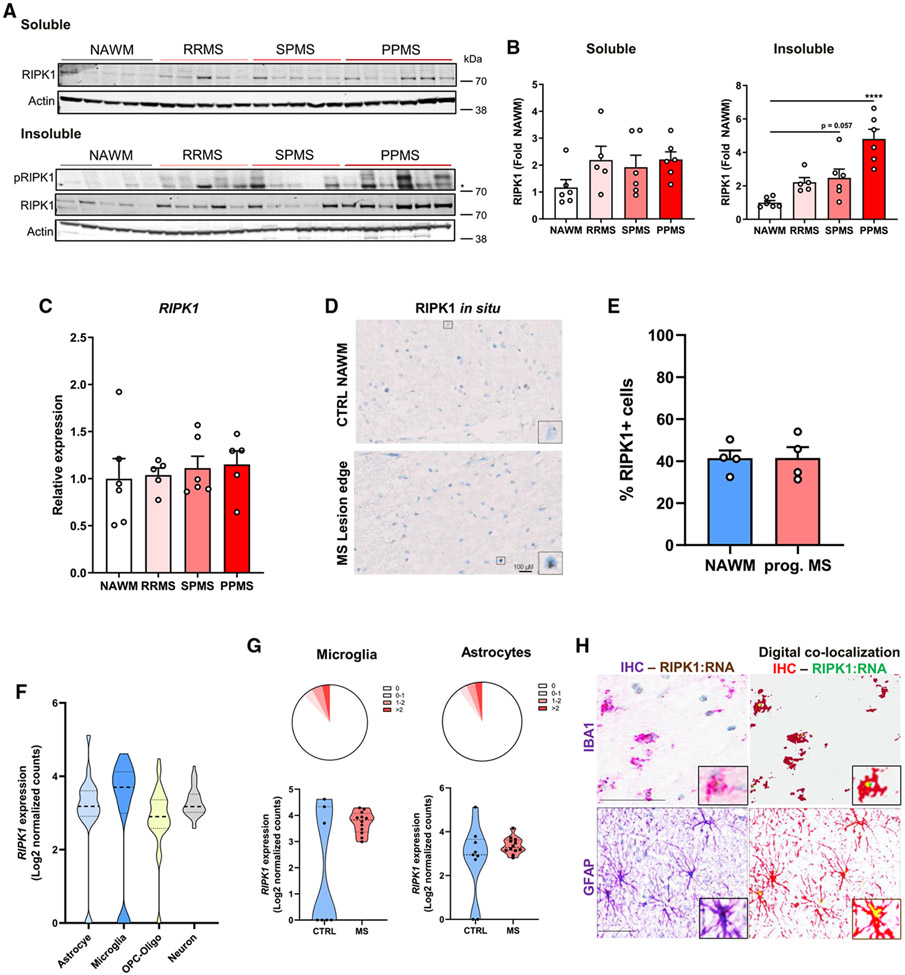
RIPK1 protein levels are elevated in progressive forms of MS (A) Protein levels assessed by immunoblotting soluble (TBS/Triton) and insoluble (RIPA/urea) brain lysates from control (CTRL) and MS patients; *non-specific band. (B) Quantification of blots in (A) normalized to NAWM control (n = 5 RRMS; n = 6 NAWM, SPMS, and PPMS samples). (C) qRT-PCR analysis of *RIPK1* expression in control and MS patient samples from (B). (D) RIPK1 *in situ* hybridization (ISH) of CTRL and progressive MS (prog. MS) brains (perilesion area). The inset depicts RIPK1+ ISH as brown dots. Scale bar: 100 μM. (E) Quantification of RIPK1+ ISH cells (n = 4 per group). 2–3 areas of similar size were quantified per sample, including lesion area in prog. MS samples. (F) Pseudo-bulk analysis of *RIPK1* expression in astrocytes, microglia, oligodendrocytes and OPCs, and neurons from control and MS patients from an RNA-seq database (Schirmer et al., 2019). (G) Single-cell *RIPK1* expression represented by read count distribution in microglia and astrocytes in control and MS patients (top), with violin plots (bottom) depicting pseudo-bulk analysis expression from (F) in individuals (n = 7–9 control, n = 12 MS). (H) ISH and immunohistochemistry (IHC) staining of prog. MS brains (perilesion area) depicting RIPK1+ ISH as brown dots and IBA1+ microglia/macrophages or GFAP+ astrocytes in purple. Digital co-localization of RIPK1 and IBA1 or GFAP is depicted in pseudo-yellow in the analysis image (inset), where RIPK1+ ISH is green and IBA1+ or GFAP+ IHC is red. Scale bar: 50 μM. Error bars represent mean ± SEM. Images are representative of 4 NAWM and 4 prog. MS samples (D and H). One-way ANOVA with Tukey post hoc test was performed (B). ****p < 0.0001. See also Figure S1 and Table S1.

To explore the role of RIPK1 in MS, we examined RIPK1 expression in post-mortem brain samples from patient-derived white matter lesions and normal-appearing white matter (NAWM) in control subjects. RIPK1 protein levels were increased in MS brains compared with NAWM controls, especially in the insoluble protein fraction (Figure S1A). To determine whether RIPK1 is increased in specific MS stages, we assessed post-mortem samples from RRMS or progressive MS (SPMS and PPMS). Although RIPK1 reactivity was increased in the Tris-buffered saline (TBS)/Triton-soluble fraction in MS brain samples compared with NAWM controls, RIPK1 expression was significantly elevated in the insoluble (radioimmunoprecipitation assay [RIPA]/urea soluble) fraction of PPMS samples (Figures 1A and 1B). Using a phospho-RIPK1 (pRIPK1) antibody to examine RIPK1 activation status, we observed pRIPK1 was associated with all forms of MS but especially elevated in PPMS (Figure 1A). Interestingly, elevated RIPK1 levels in PPMS patients correlated with the loss of myelin basic protein (MBP) expression, suggesting increased demyelination may be linked to RIPK1 activation (Figures S1A and S1B). We used the sensitivity of the Meso Scale Discovery (MSD) platform to assess RIPK1 activation and expression levels, demonstrating increased pRIPK1 and total RIPK1 levels in progressive MS compared with RRMS and NAWM controls (Figures S1C and S1D). The downstream necroptosis executioner MLKL is phosphorylated to induce its oligomerization and activation (Wang et al., 2014). We observed a trend of increased MLKL activation and a significant increase in MLKL levels in progressive MS brains (Figures S1E and S1F). Caspase-8 levels were increased in MS samples compared with NAWM controls, but there was no significant distinction across the various MS subtypes (Figures S1G and S1H). Thus, although caspase-8 expression appears elevated in MS, these data clearly demonstrate that RIPK1 is activated in progressive MS, suggesting RIPK1 may have a key and unique regulatory role in progressive MS.

To determine whether increases in RIPK1 result from increased transcription, we assessed *RIPK1* mRNA levels. We observed no changes in *RIPK1* RNA levels in MS brain samples compared with NAWM controls by qPCR or *in situ* hybridization (ISH) analysis (Figures 1C-1E), suggesting increased protein levels in PPMS are regulated post-translationally. To elucidate the cell types that express RIPK1 in MS lesions, we used single-nucleus RNA-seq from control and MS patients (Schirmer et al., 2019), which demonstrated *RIPK1* is ubiquitously expressed in the CNS (Figure 1F). We observed similar expression in control and MS patients in microglia and astrocytes, key cell types contributing to CNS homeostasis and neuroinflammation (Figure 1G). To confirm these results, we used a dual ISH and immunohistochemistry approach to validate *RIPK1* mRNA is expressed in IBA1+ microglia and GFAP+ astrocytes at similar levels in NAWM controls and progressive MS samples (Figures 1H and S1I-S1K). Although microglia migrate near the lesion edge, *RIPK1* mRNA did not appear increased in this area (Figure S1I). Our results suggest RIPK1 signaling may be activated post-translationally in microglia and/or astrocytes and contribute to the overall increase in RIPK1 protein levels in progressive MS. This is consistent with emerging evidence suggesting MS progression partly results from an altered innate immune response within the CNS and in particular dysregulation of microglial homeostasis (Baecher-Allan et al., 2018; Ponath et al., 2018b; Voet et al., 2019; Zrzavy et al., 2017).

### RIPK1 signaling in microglia is pro-inflammatory and deleterious

**Figure 2.**
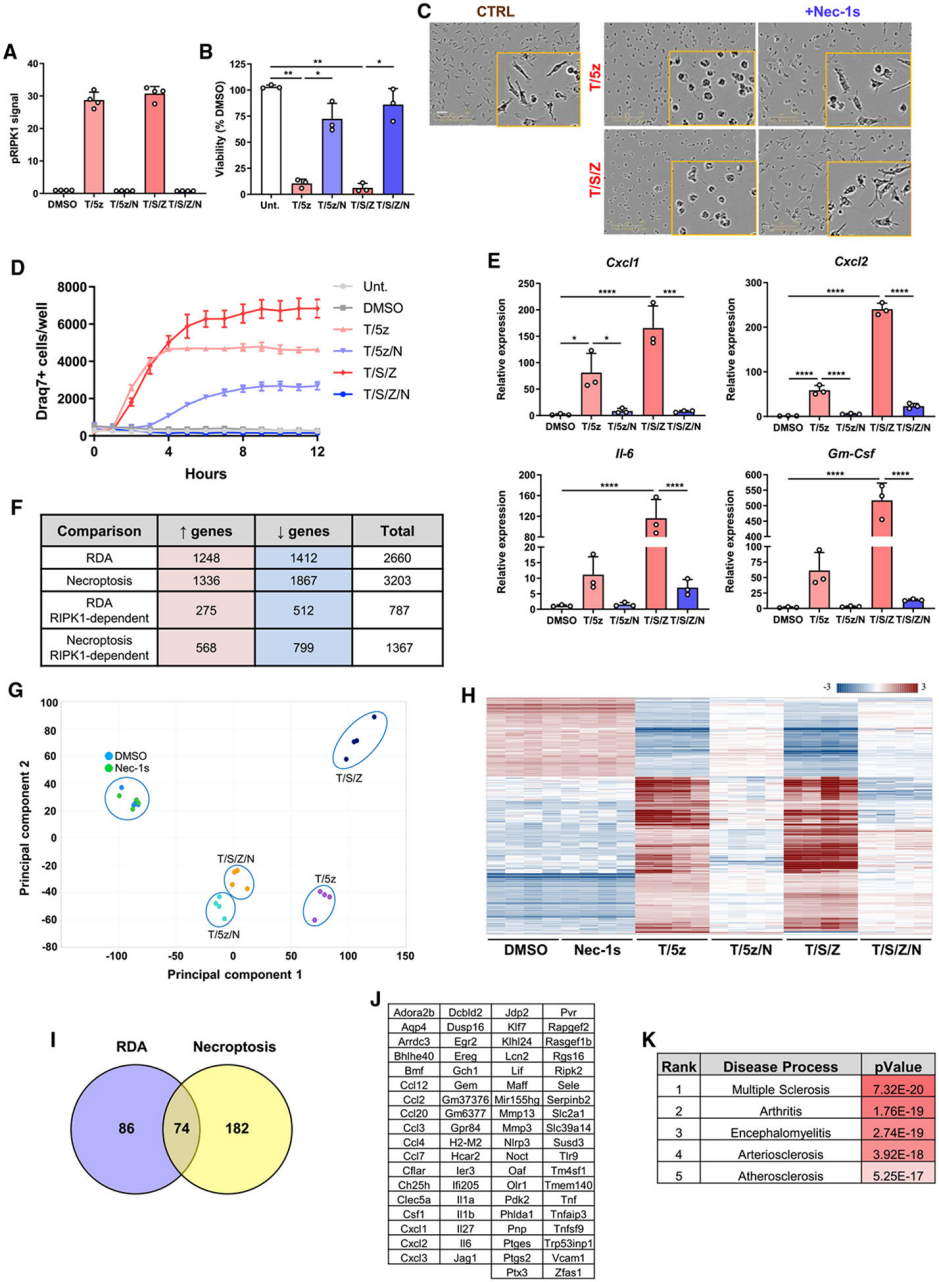
RIPK1 kinase activity regulates pro-inflammatory signaling and cell death in murine microglia *in vitro* (A) MSD assay quantifying pRIPK1 levels in microglia treated for 2 h. (B) Viability of microglia stimulated for 20 h (n = 3). (C) Representative images depicting microglial morphology 20 h after treatment. Scale bar: 100 μM. (D) Quantification of DRAQ7+ microglia imaged in the IncuCyte Zoom system. (E) Relative gene expression in microglia treated for 4 h (n = 3). (F) RNA-seq data in microglia treated with T/5z (RDA) or T/S/Z (necroptosis) for 4 h with or without Nec-1s (RIPK1-dependent), comparing upregulated and downregulated genes (cutoff ∣FC∣ > 1.5, p < 0.05). (G) PCA of RNA-seq microglia (n = 4). (H) Hierarchical heatmap clustering of RIPK1-kinase-dependent genes. (I) Overlap of RIPK1-dependent genes (cutoff ∣FC∣ > 2, p < 0.05). (J) List of overlapping RIPK1-dependent genes from (I). (K) Top 5 diseases associated with genes in (J) from the ToppGene database. Error bars represent mean ± SD. Data depict technical replicates and are representative of 2 (A) or 3 (D) independent experiments. One-way ANOVA with Tukey post hoc test was performed (B and E). *p < 0.05, **p < 0.01, ***p < 0.001, ****p < 0.0001. T, TNF; S, Smac; Z, zVAD; N, Nec-1s. See also Figures S2 and S3.

To date, RIPK1 has been extensively studied in the context of the immune response. Microglia regulate the CNS innate immune response, and RIPK1 can be activated by various stimuli. However, the outcome of the RIPK1 response is unclear. To better understand the consequences of RIPK1 activation and signaling in microglia, we used two stimulations to induce RIPK1 kinase activity. Tumor necrosis factor (TNF, further shortened to T) stimulation with 5z-7 (5z), a TAK1 kinase inhibitor, can induce RDA (Geng et al., 2017), whereas TNF stimulation with Smac mimetic (S) and the pan-caspase inhibitor zVAD (Z) (Wang et al., 2008) leads to necroptosis. After 2 h, T/5z or T/S/Z stimulation upregulated pRIPK1, whereas the signal was abrogated by Nec-1s (N), a small-molecule RIPK1 kinase inhibitor (Figure 2A; Degterev et al., 2005, 2008). Furthermore, both stimulations induced significant cell death, which was attenuated in the presence of Nec-1s (Figure 2B). Cell death occurred within 6 h and was accompanied by morphological changes, whereas T/5z led to RIPK1-kinase-independent apoptosis with delayed induction kinetics (Figures 2B-2D). These data indicate RIPK1 activation in microglia can lead to both apoptosis and necroptosis.

Recently, RIPK1 kinase activity has been implicated in both transcriptional regulation of cell-death-independent cytokine/chemokine production and a pro-inflammatory transcriptional program that accompanies cell death (Najjar et al., 2016; Zhu et al., 2018). To assess whether microglial RIPK1 kinase activation regulates an inflammatory gene profile, we examined transcriptional changes in these cells. T/5z or T/S/Z stimulation upregulated pro-inflammatory gene expression and protein secretion in a RIPK1-kinase-dependent manner (Figures 2E and S2A), demonstrating microglial RIPK1 kinase activation can lead to both inflammatory response and cell death. However, because RIPK1 kinase activation leads to rapid microglial death, we compared the temporal sequence of these two processes. The immediate transcriptional response was largely independent of RIPK1 kinase activity, but as the cell death machinery is engaged, the inflammatory response becomes driven by RIPK1 kinase (Figures S2B and S2C).

To uncouple inflammation from cell death, we used two additional stimulations to activate pRIPK1: TNF with Smac (T/S), which can lead to apoptosis (Wang et al., 2008), and TNF and zVAD (T/Z), a weaker inducer of necroptosis (Holler et al., 2000). In microglia, both stimulations increased RIPK1 kinase activity, and this response was blocked by Nec-1s (Figure S2D). T/S induced minimal cell death, whereas T/Z-induced necroptosis occurred at lower levels and with slower kinetics compared with T/5z or T/S/Z stimulation (Figures S2E and S2F). Both T/S and T/Z treatment upregulated the transcription and secretion of inflammatory chemokines (Figures S2G and S2H), whereas TNF alone induced transient, low-level RIPK1 activation but no cell death (Figures S2I and S2J). Furthermore, TNF-induced transcriptional upregulation of inflammatory chemokines was independent of RIPK1 kinase (Figure S2K). Because both T/S- and T/Z-induced transcriptional responses occurred before cell death, these data suggest RIPK1 kinase activity can regulate a death-independent transcriptional response.

To clarify the role of RIPK1-kinase-dependent transcriptional signaling in microglia, we used RNA-seq to profile the transcriptome of T/5z- and T/S/Z-treated microglia. Both stimuli induced a robust response that depended partially on RIPK1 kinase activity (Figure 2F). Although the kinase-independent response was mediated by TNF, Nec-1s stimulation had no effect, whereas T/5z and T/S/Z treatment broadly altered the microglial transcriptome compared with DMSO control (Figures 2G and 2H). The top putative upstream regulators included transcription factors of the Sp1, nuclear factor κB (NF-κB) and AP-1 families (Figures S3A-S3C). We identified more than 300 genes modulated in a RIPK1-kinase-dependent manner in response to either stimulation, but the transcriptional response downstream of these two stimuli did not fully overlap, suggesting the RIPK1-driven transcriptional response is context specific (Figure 2I). The core RIPK1-kinase-dependent genes consisted of an immune/inflammatory-related signature, demonstrating the harmful inflammatory nature of RIPK1 kinase activation in microglia (Figure S3D). Importantly, the gene signature consisted of various cytokines and chemokines elevated in the serum or cerebrospinal fluid (CSF) of MS patients, including CXCL1, CCL2, CCL3, CCL4, and interleukin-6 (IL-6) (Figure 2J; Göbel et al., 2018; Khaibullin et al., 2017; Soleimani et al., 2019; Stampanoni Bassi et al., 2018). Genes involved in lipid metabolism or the transcription factor *Bhlhe40* and microRNA *mir155hg*, both of which have been implicated as detrimental in a mouse model of MS (Lin et al., 2014; Murugaiyan et al., 2011; O’Connell et al., 2010), were upregulated in a RIPK1-kinase-dependent manner (Figure S3E). Using a gene-disease association database, we identified MS and inflammatory disorders as the diseases most significantly associated with the core RIPK1-kinase-dependent gene signature (Figure 2K). These results suggest microglial RIPK1 activation drives a transcriptional program that may modulate neuroinflammation and contribute to MS pathology and progression.

### RIPK1 signaling in astrocytes is pro-inflammatory but does not induce cell death

**Figure 3.**
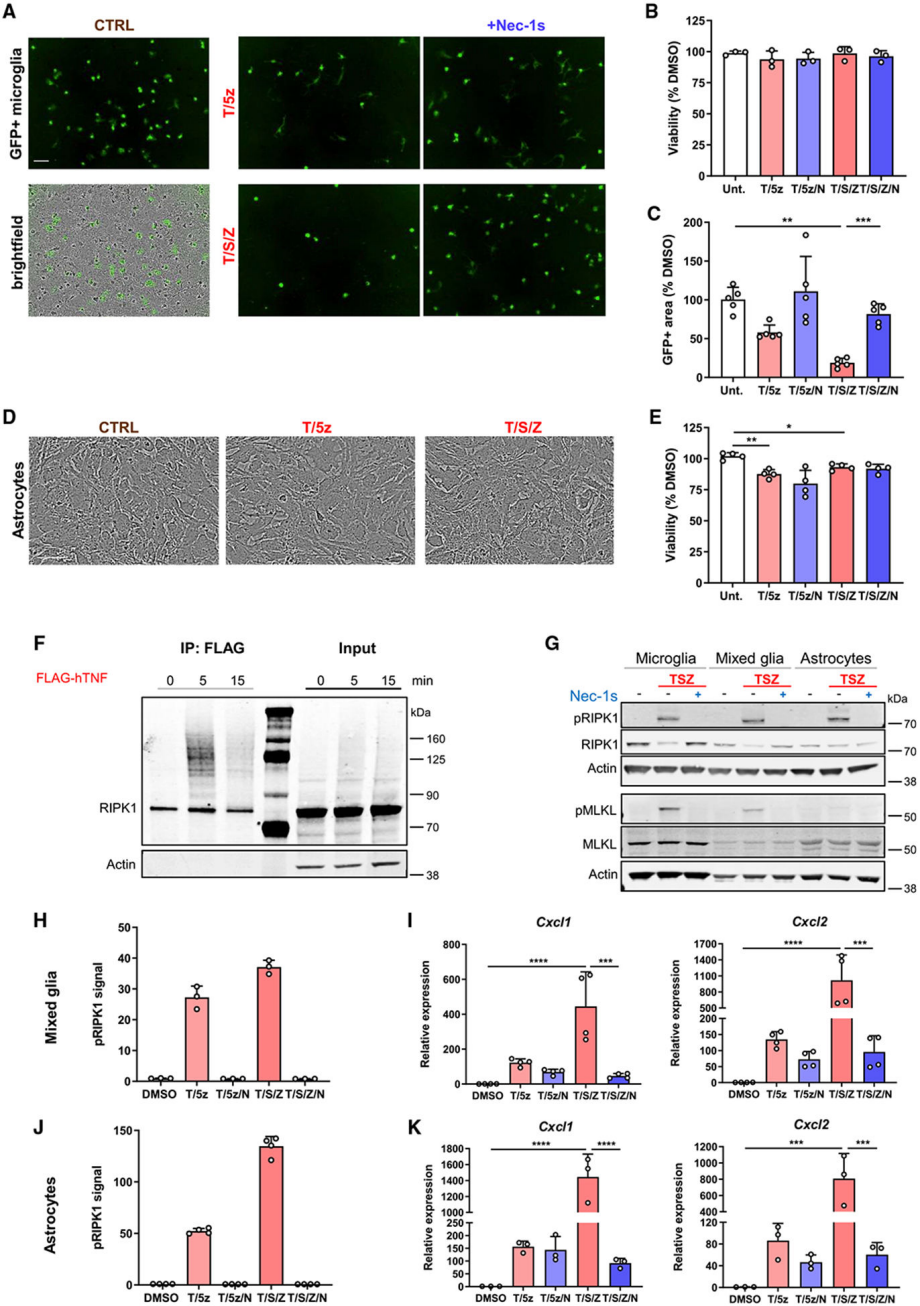
RIPK1 kinase activation mediates pro-inflammatory signaling, but not cell death, in murine mixed glia and astrocytes *in vitro* (A) Representative images depicting GFP+ microglia seeded on mixed glial culture and treated for 48 h. Scale bar: 100 μM. (B) Viability of mixed glia stimulated for 20 h (n = 3). (C) Quantification of GFP+ microglia in mixed glial culture measured as GFP+ area normalized to DMSO control (n = 5). (D) Representative images depicting astrocytes 20 h after treatment. Scale bar: 100 μM. (E) Viability of astrocytes stimulated for 20 h (n = 4). (F) Protein expression in astrocytes assessed by immunoblotting after stimulation with FLAG-tagged human TNF and immunoprecipitation with anti-FLAG. (G) Protein expression assessed by immunoblotting after 2 h stimulation in microglia, mixed glia, and astrocytes. (H–K) MSD quantification of pRIPK1 in mixed glia (H) or astrocytes (J) treated for 2 h. Relative gene expression in mixed glia (I) or astrocytes (K) treated for 4 h (n = 4 mixed glia, n = 3 astrocytes). Error bars represent mean ± SD. Data depict technical replicates and are representative of 2 independent experiments (F–H and J). One-way ANOVA with Tukey post hoc test was performed (C, E, I, and K). *p < 0.05, **p < 0.01, ***p < 0.001, ****p < 0.0001. See also Figure S4.

Although RIPK1 is expressed in microglia, there are significant levels present in other CNS cells, such as astrocytes (Figures 1F-1H). These glial cells are critical for CNS function, and their dysfunction has been suggested to contribute to MS pathophysiology (Lassmann, 2019; Ponath et al., 2018b; Voet et al., 2019). Furthermore, microglia work in concert with other CNS cells to maintain homeostasis. To explain the role of microglial RIPK1 signaling in the presence of other cell types, we used a mixed glial culture system consisting of astrocytes, endothelial cells, OPCs and microglia, and exogenously applied, fluorescently labeled microglia. The mixed cultures were resistant to T/5z- and T/S/Z-induced cell death, but when we specifically examined GFP+ microglia, we observed loss in a RIPK1-kinase-dependent manner (Figures 3A-3C). These data suggest changes in mixed glial viability were not detected because only a subset of cells, the microglia, undergo cell death. When we stimulated astrocytes with T/5z and T/S/Z, we observed no RIPK1-kinase-dependent decrease in viability (Figures 3D and 3E). To ensure RIPK1 signaling in astrocytes was intact, we confirmed RIPK1 is expressed and recruited to the TNF receptor after brief TNF stimulation. RIPK1 was rapidly recruited to TNFR1 and heavily ubiquitinated, and by 15 min, the levels of RIPK1 at the receptor had returned to baseline (Figure 3F). Furthermore, microglia, astrocytes, and mixed glial cells underwent RIPK1 auto-iksphorylation at S166 upon T/S/Z stimulation, and this response was abrogated by Nec-1s (Figure 3G). MLKL appeared to be expressed at lower levels in astrocytes compared with microglia. Consequently, T/S/Z stimulation induced phospho-MLKL (pMLKL) in a RIPK1-kinase-dependent manner in microglia, but not astrocytes (Figure 3G). In mixed glial cultures, pMLKL was weakly activated downstream of T/S/Z stimulation, suggesting the detected signal comes from microglia present in the mixed culture.

Although both mixed glia and astrocytes are capable of pRIPK1 induction upon T/5z or T/S/Z stimulation (Figures 3G, 3H, and 3J), unlike microglia, the astrocytes do not undergo RIPK1-kinase-dependent cell death. Similarly, T/S and T/Z stimulation in astrocytes increased pRIPK1 levels but did not induce RIPK1-dependent cell death (Figures S4A and S4B). To determine the role of RIPK1 signaling in astrocytes, we examined whether RIPK1 regulates a transcriptional response in the absence of cell death. In a mixed glial culture, which contains a subset of microglial cells, T/5z or T/S/Z treatment induced a RIPK1-kinase-dependent chemokine response (Figure 3I). To confirm this transcriptional response did not occur exclusively in microglia, we treated isolated astrocytes with T/5z or T/S/Z. Both stimuli strongly increased chemokine expression in a RIPK1-kinase-dependent manner (Figure 3K). In addition, T/S and T/Z stimulation in astrocytes upregulated inflammatory gene expression in a RIPK1-kinase-dependent manner, whereas the TNF-induced response was independent of RIPK1 kinase activity (Figure S4C). In addition, T/S/Z-induced IL-6 secretion required RIPK1 kinase activity (Figure S4D). To confirm the role of RIPK1 in this transcriptional response, we used small interfering RNA (siRNA) to reduce *Ripk1* expression before stimulating astrocytes. Following *Ripk1* knockdown, we observed a blunted inflammatory response upon T/5z or T/S/Z treatment (Figure S4E). Our data suggest RIPK1 activity regulates an inflammatory transcriptional program in both microglia and astrocytes *in vitro*, but in astrocytes this program is uncoupled from RIPK1-mediated cell death.

Recent studies suggest necroptosis can activate inflammatory gene expression in a cell-autonomous manner and that the second wave of cytokine production involves NF-κB signaling downstream of RIPK1 and RIPK3 activation (Zhu et al., 2018). To determine whether similar mechanisms regulate inflammatory transcriptional programs in microglia and astrocytes, we assessed the roles of RIPK1, RIPK3, and NF-κB using a pharmacological approach. In both cell types, we found T/S/Z-induced transcriptional upregulation of chemokines depended on inhibitor of nuclear factor-κB kinase (IKK)/NF-κB signaling, as assessed by the IKKα/β inhibitor TPCA-1, and the kinase activity of RIPK3 (Figures S4F and S4G). Thus, in addition to RIPK1 kinase activation, the T/S/Z-induced transcriptional program in microglia and astrocytes requires RIPK3 kinase activity and NF-κB signaling.

To definitively assess the RIPK1-kinase-driven transcriptional response in the absence of cell death, we used MLKL-deficient cells (Murphy et al., 2013). As expected, *Mlkl*^−/−^ microglia and astrocytes were resistant to T/S/Z-induced necroptosis. Conversely, T/5z-induced apoptosis occurred independently of both RIPK3 kinase activity and MLKL in microglia, whereas astrocytes were resistant to RDA (Figures S4H and S4J). Importantly, we observed RIPK1-kinase-dependent regulation of a transcriptional response in MLKL-deficient cells. In microglia, this inflammatory response was independent of RIPK3 kinase activity, whereas in astrocytes, it was partially dependent on RIPK3 (Figures S4I and S4K). These data demonstrate RIPK1 kinase activation can drive a transcriptional response in microglia and astrocytes independently of cell death.

### RIPK1 signaling in astrocytes drives a disease-relevant gene signature

**Figure 4.**
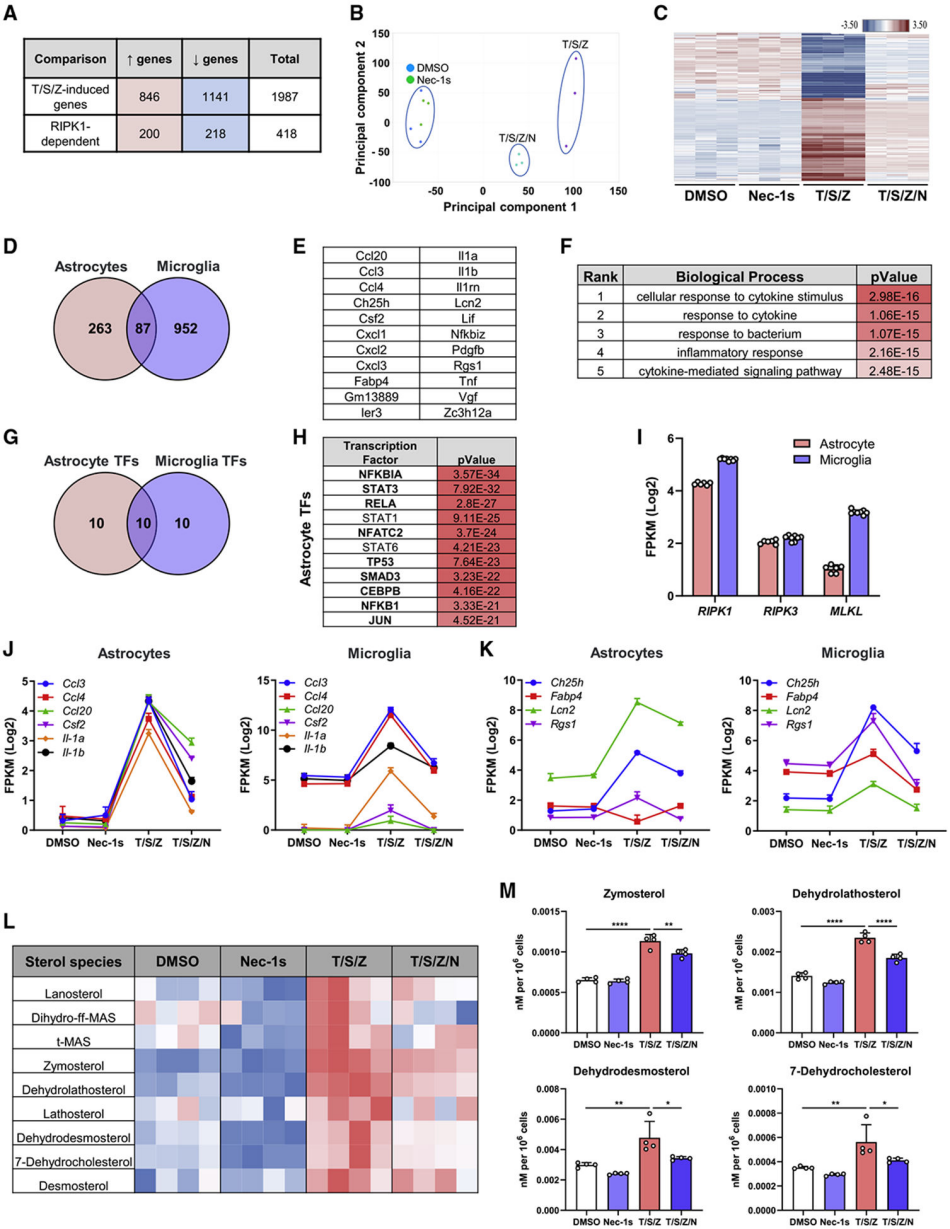
RIPK1 kinase activity regulates a pro-inflammatory transcriptional program in murine astrocytes *in vitro* (A) RNA-seq in astrocytes comparing upregulated and downregulated genes with T/S/Z or T/S/Z/N (RIPK1 dependent) stimulation (cutoff ∣FC∣ > 1.5, p < 0.05). (B) PCA of astrocytes (n = 3) treated for 4 h. (C) Hierarchical heatmap clustering of RIPK1-kinase-dependent genes. (D) Overlap of RIPK1-dependent genes from T/S/Z-stimulated microglia and astrocytes (cutoff ∣FC∣ > 1.5, p < 0.05). (E) List of overlapping RIPK1-dependent genes from (D), with cutoff ∣FC∣ > 2. (F) Gene Ontology terms for biological processes from ToppGene Suite for genes in (E). (G) Overlap of upstream transcriptional regulators predicted in Ingenuity Pathway Analysis (IPA) for genes in (D). (H) Top transcriptional regulators in astrocytes from (G), with overlapping transcription factors in bold. (I) Gene expression in astrocytes and microglia (n = 6 astrocytes, n = 8 microglia for DMSO and Nec-1s samples). (J and K) Expression of individual genes involved in inflammatory (J) or lipid and metabolism (K) signaling. (L) Heatmap of sterol profile from astrocytes treated for 24 h (n = 4). (M) Concentrations of altered sterol species in astrocytes treated for 24 h (n = 4). Error bars represent mean ± SD. One-way ANOVA with Tukey post hoc test was performed. *p < 0.05, **p < 0.01, ****p < 0.0001. See also Figure S5.

Our data suggest astrocytes represent a cell type in which RIPK1 kinase activity regulates neuroinflammatory responses in the absence of cell death. To examine the role of RIPK1 in transcriptional regulation in astrocytes, we performed RNA-seq. T/S/Z stimulation induced a strong transcriptional alteration that partially depended on RIPK1 (Figure 4A). The RIPK1-kinase-dependent response was visualized by principal-component analysis (PCA) and heatmap clustering (Figures 4B and 4C). We identified an overlap of 87 RIPK1-kinase-dependent genes between microglia and astrocytes. A subset of these highly induced genes (fold change [FC] > 2) consisted of primarily cytokines and chemokines, and Gene Ontology analysis revealed a strong immune/inflammatory signature (Figures 4D-4F). Putative upstream regulators in astrocytes included members of the NF-κB and AP-1 families and a 50% overlap with the top 20 putative microglial transcription factors (Figures 4G and 4H). To explain the differential susceptibility of microglia and astrocytes to necroptosis, we examined *Ripk1, Ripk3*, and *Mlkl* expression in our RNA-seq datasets. Although *Ripk1* and *Ripk3* levels were similar, *Mlkl* expression was lower in astrocytes compared with microglia, consistent with protein levels (Figures 3G and 4I). Thus, astrocytes may be resistant to RIPK1-kinase-mediated necroptosis because of low MLKL expression. The overlapping inflammation-related signature suggests RIPK1 signaling in CNS cells may regulate various cytokines and chemokines implicated in MS pathogenesis (Figure 4J; Göbel et al., 2018; Jafarzadeh et al., 2014; Khaibullin et al., 2017; Li et al., 2017; Ofengeim et al., 2017; Seppi et al., 2014; Soleimani et al., 2019). Genes involved in lipid signaling and metabolism previously linked to MS and its progression were modulated in a RIPK1-kinase-dependent manner in astrocytes and microglia (Figure 4K; International Multiple Sclerosis Genetics Conssortium(IMSGC), 2010; Al Nimer et al., 2016; Bove et al., 2019; Forwell et al., 2016; Keyhanian et al., 2019; Lee and Bou Dagher, 2016). Because our RNA-seq data identified multiple genes linked to cholesterol signaling and function, we assessed the role of RIPK1 in modulating lipid metabolism by performing lipidomic analysis of sterols in astrocytes. Astrocytes were chosen so that we could uncouple cell death and lipid homeostasis. T/S/Z-activated astrocytes significantly upregulated various sterol intermediates in a RIPK1-kinase-dependent manner (Figures 4L and 4M), suggesting a disruption of the homeostatic cholesterol biosynthesis pathway. These results show RIPK1 kinase activation regulates a common, disease-relevant signaling cascade in astrocytes and microglia in a cell-autonomous manner.

### RIPK1 kinase activation is detrimental in human cells

To investigate the susceptibility of other CNS cells to RIPK1-kinase-dependent death, we examined neurons and OPCs. Similar to astrocytes, these cells did not undergo RIPK1-kinase-dependent death, although OPCs appear sensitive to RIPK1-independent apoptosis (Figures S5A and S5B). To determine the consequence of RIPK1 activation in human-derived cells, we stimulated human monocyte-derived macrophages and found them to be susceptible to T/5z- and T/S/Z-induced death (Figure S5C). Furthermore, RIPK1 can be activated in both induced pluripotent stem cell (iPSC)-derived microglia and human fetal-derived astrocytes upon T/S/Z stimulation (Figures S5D and S5E). Similar to murine cells, human microglia, but not astrocytes, were susceptible to necroptosis (Figures S5F and S5G). Previous RNA-seq data have shown human fetal-derived astrocytes express lower levels of MLKL compared with microglia (Figure S5H; Zhang et al., 2016), consistent with the lack of necroptotic response in astrocytes. Interestingly, T/S/Z induced inflammatory gene transcription in a RIPK1-kinase-dependent manner in human microglia but to a lesser extent in astrocytes (Figures S5I and S5J). To elucidate signaling unrelated to cell death in human astrocytes, we performed RNA-seq and identified a RIPK1-kinase-dependent transcriptional signature related to interferon (IFN) signaling and antiviral response (Figures S5K-S5M). These data extend our findings from murine cells to disease-relevant human-derived glia and show RIPK1 kinase signaling in astrocytes and microglia may drive MS pathogenesis.

### RIPK1 kinase activation is detrimental to oligodendrocytes in a non-cell-autonomous manner

**Figure 5.**
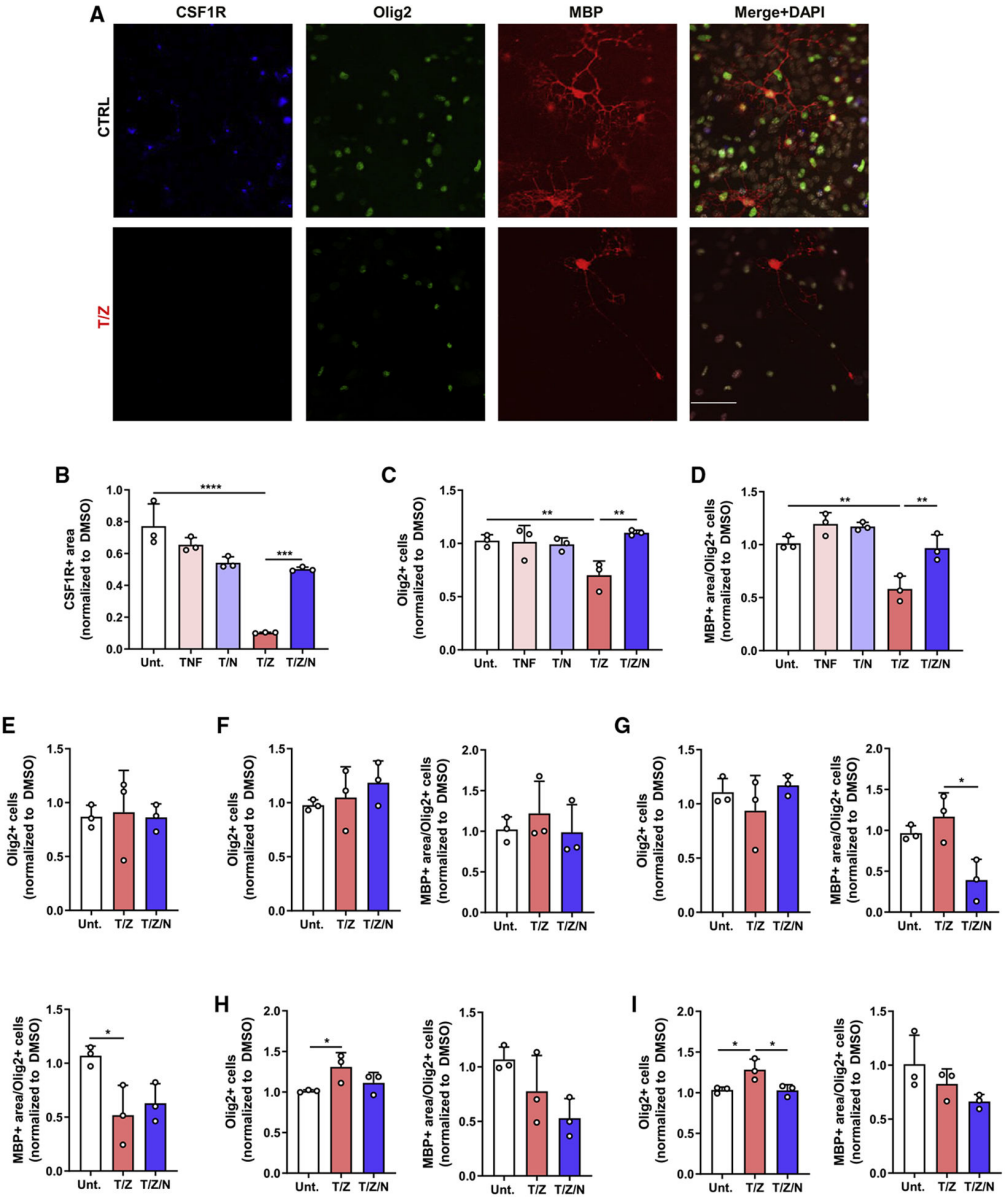
RIPK1 kinase activation in microglia and astrocytes mediates deleterious non-cell-autonomous signaling *in vitro* (A) Representative images depicting OPCs seeded on mixed glial cells, treated for 72 h, and stained with indicated antibodies. Scale bar: 50 μM. (B–D) Quantification of CSF1R+ area (B), Olig2+ cells (C), and MBP+ area (D) from OPCs co-cultured with mixed glial cells and treated for 72 h (n = 3). (E–I) Quantification of Olig2+ cells and MBP+ area from OPCs cultured with mixed glia (E), astrocyte (H), or microglia (I) conditioned media or OPCs co-cultured with astrocytes (F) or microglia (G) and treated for 48 h (E) or 72 h (F–I) as outlined (n = 3). Error bars represent mean ± SD. One-way ANOVA with Tukey post hoc test was performed. *p < 0.05, **p < 0.01, ***p < 0.001, ****p < 0.0001.

Although OPCs were resistant to RIPK1-kinase-mediated death, we wanted to assess whether RIPK1 activation affected OPCs in a non-cell-autonomous manner. We added OPCs to a mixed glial culture and stimulated the cells with a RIPK1-activating stimulus. Although TNF stimulation had limited effect on viability, T/Z stimulation significantly reduced CSF1R+ microglia in a RIPK1-dependent manner (Figures 5A and 5B). Immunofluorescence staining for Olig2 indicated OPCs and oligodendrocytes were reduced in a RIPK1-kinase-dependent manner upon stimulation with T/Z, but not TNF, in the co-culture system. OPC maturation and myelinating oligodendrocytes were also decreased in a RIPK1-kinase-dependent manner upon T/Z treatment, as quantified by MBP+ area (Figures 5A, 5C, and 5D). These results suggest RIPK1 activation in microglia and/or astrocytes is harmful and reduces OPC differentiation and myelinating capacity. To determine whether these effects are mediated by secreted factors, we added mixed glia conditioned media to OPCs. The number of Olig2+ cells was not altered, suggesting secreted factors by mixed glial cells are insufficient to alter oligodendrocyte differentiation and survival (Figure 5E). Because microglia and astrocytes upregulate RIPK1 kinase activity, we wanted to determine which cell type mediates the deleterious effects on OPCs. However, we did not detect reductions in Olig2+ cells or MBP+ area when OPCs were co-cultured with T/Z-stimulated astrocytes or microglia or with their conditioned media (Figures 5F-5I). These data show direct cell-cell contact with both microglia and astrocytes is required for deleterious RIPK1-kinase-mediated effects on OPCs. This suggests RIPK1 activation in glial cells can affect myelination through direct interactions with OPCs and regulation of cytokine and chemokine synthesis may recruit peripheral immune cells in a disease context.

### RIPK1 kinase inhibition attenuates EAE severity and disease progression

**Figure 6.**
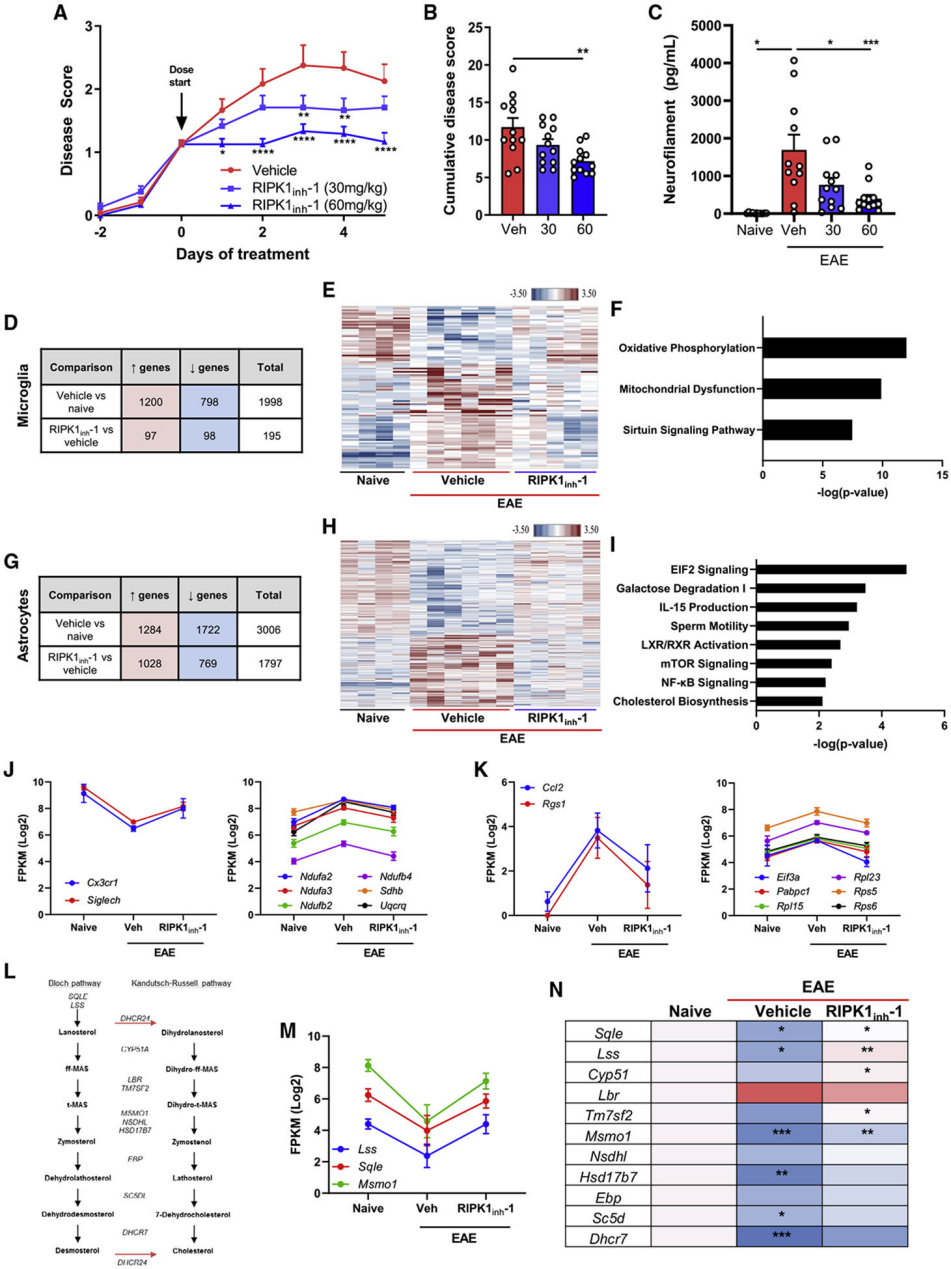
RIPK1 kinase inhibition attenuates clinical disease progression and suppresses microglial and astrocyte signaling pathways in a murine model of MS (A) Mean disease scores for vehicle (0.5% methylcellulose, twice a day [BID]) and RIPK1 kinase inhibitor groups (RIPK1_inh_-1, 30 or 60 mg/kg BID) in C57BL/6 EAE mice (n = 12 mice/group). (B) Cumulative disease scores for each mouse are depicted. (C) Neurofilament heavy levels measured in the plasma of naive or EAE-induced mice at sacrifice (n = 11 naive and vehicle, n = 12 RIPK1_inh_-1). (D–H) RNA-seq data in microglia (D) and astrocytes (G) isolated from naive or EAE-induced mice depicting upregulated and downregulated genes (cutoff ∣FC∣ 1.5, p < 0.05; n = 4 naive, n = 6 EAE-vehicle [20% Captisol], and n = 5 EAE-RIPK1_inh_-1 [50 mg/kg BID] mice). Hierarchical heatmap clustering of RIPK1-kinasedependent genes in microglia (E) and astrocytes (H). Top pathways predicted from IPA for RIPK1-kinase-dependent genes in microglia (F) and astrocytes (I). (J–M) Gene expression levels in RNA-sequenced microglia (J) and astrocytes (K and M); *Ccl2* p = 0.072 (EAE-vehicle versus EAE-RIPK1_inh_-1), all other genes p < 0.05. (L) Diagram of genes and sterol intermediates in the cholesterol biosynthesis pathway. (N) Heatmap of cholesterol biosynthesis genes from RNA-sequenced astrocytes, normalized to naive mice, with significant changes for naive versus EAE-vehicle and EAE-vehicle versus EAE-RIPK1_inh_-1 shown. Error bars represent mean ± SEM. One-way ANOVA with Tukey post hoc test (B and C) or two-way ANOVA with Dunnett test (A) was performed. *p < 0.05, **p < 0.01, ***p < 0.001, ****p < 0.0001. See also Figure S6.

Having established the deleterious role of RIPK1 activation *in vitro*, we investigated its role in a murine model of MS. Previous groups have used RIPK1 kinase inhibitors prophylactically to reduce disease scores in the C57BL/6 EAE model (Harris et al., 2019; Ofengeim et al., 2015; Yoshikawa et al., 2018; Zhang et al., 2019). Therefore, we tested RIPK1 inhibition after disease onset. Therapeutic administration of a CNS-penetrant RIPK1 kinase inhibitor was efficacious in reducing EAE disease severity in a dose-dependent manner, as assessed by mean and cumulative disease scores (Figures 6A and 6B). Therapeutic administration of the 60 mg/kg dose halted clinical disease score increases and reduced plasma neurofilament levels, a marker of axonal and neuronal damage (Figure 6C; Dujmovic, 2011; Petzold, 2005). Therefore, RIPK1 kinase inhibition can attenuate disease severity in this EAE model.

Because the C57BL/6 EAE model has a weaker, monophasic disease course (Berard et al., 2010; Bittner et al., 2014) and we observe RIPK1 upregulation, particularly in progressive MS brain samples (Figures 1A and 1B), we wanted to investigate the role of RIPK1 in a progressive model. We used the NOD-EAE model, in which mice endure initial attacks and remissions before entering a progressively worsening disease phase (Ignatius Arokia Doss et al., 2015; Levy et al., 2010). pRIPK1 and total RIPK1 were elevated in spinal cords of mice in the progressive disease phase (Figures S6A-S6C). To test whether RIPK1 kinase inhibition is beneficial in this model, we randomized animals into vehicle or inhibitor groups after the initial remission and relapse phase. RIPK1 inhibition significantly attenuated disease progression, as measured by mean disease score (Figure S6D). Our data indicate RIPK1 kinase inhibition is beneficial when administered therapeutically in multiple preclinical MS models.

To examine whether RIPK1 kinase signaling in astrocytes or microglia contributes to EAE, we sorted spinal cord astrocytes and microglia after 3 days of treatment with a RIPK1 inhibitor and performed RNA-seq analysis (Figure S6E). We identified RIPK1-kinase-dependent transcriptional alterations in both cell types during EAE (Figures 6D-6I, S6F, and S6G). Strikingly, only about 5% of genes differentially expressed in microglia in EAE were modulated by RIPK1 kinase inhibition, suggesting highly selective RIPK1 function in these cells. Meanwhile, in astrocytes, a quarter of differentially expressed genes were regulated in a RIPK1-kinase-dependent manner, suggesting a broader RIPK1 role in pathologic transcriptional changes in astrocytes. The top RIPK1-dependent pathways included oxidative phosphorylation and mitochondrial dysfunction in microglia and EIF2 signaling and cholesterol biosynthesis in astrocytes (Figures 6F and 6I). Genes involved in mitochondrial signaling were upregulated in a RIPK1-kinase-dependent manner in microglia during EAE. Consistent with published data, homeostatic microglial markers like *Cx3cr1* and *Siglech* were reduced during EAE (Dubbelaar et al., 2018), but RIPK1 kinase inhibition restored expression to normal levels (Figure 6J). In astrocytes, ribosome and translation initiation-related genes were increased in EAE, suggesting robust induction of translational apparatus during disease (Figure 6K). *Rgs1*, an MS risk allele, and the chemokine *Ccl2* were increased in astrocytes during EAE and reduced with RIPK1 kinase inhibition (Figure 6K). CCL2 is upregulated in astrocytes in human MS lesions (Van Der Voorn et al., 1999) and is necessary for recruitment of peripheral T cells, dendritic cells, and monocytes in EAE (Dogan et al., 2008; Kim et al., 2014). Meanwhile, multiple genes involved in cholesterol biosynthesis were reduced in a RIPK1-kinase-dependent manner in astrocytes during EAE (Figures 6L-6N). Thus, RIPK1 activity appears to contribute to mitochondrial dysfunction in microglia and altered translation and cholesterol biosynthesis in astrocytes during EAE. These data demonstrate a role for RIPK1 in microglia and astrocytes *in vivo* and suggest RIPK1 kinase inhibition in an MS disease model may be beneficial through various modes of action.

## DISCUSSION

Here, we demonstrate RIPK1 is activated and its expression is increased in progressive MS patients. Furthermore, RIPK1 kinase activation in microglia and astrocytes *in vitro* drives an inflammatory transcriptional response with harmful non-cell-autonomous consequences to oligodendrocytes and translational relevance to MS pathology. Our results demonstrate the importance of RIPK1 kinase activation to neuroinflammation and MS progression. In line with previous findings regarding RIPK1 activation in MS (Ofengeim et al., 2015), we found RIPK1 is auto-phosphorylated (pS166) and its expression is increased, particularly in the insoluble protein fraction. Importantly, we show RIPK1 is activated in post-mortem progressive MS samples, pointing to a key role for RIPK1 activation in disease progression. Studies have demonstrated microglia and astrocytes are key contributors to the neuroinflammatory environment and progression of MS (Lassmann, 2019; Ponath et al., 2018b; Voet et al., 2019), and we confirmed *RIPK1* expression in microglia and astrocytes from post-mortem progressive MS samples. Thus, RIPK1 activation in astrocytes or microglia may contribute to MS progression.

As suggested previously (Kim and Li, 2013), we found that RIPK1 kinase activation in murine microglia, but not astrocytes, neurons, or OPCs, drives RIPK1-kinase-mediated death *in vitro*. RIPK1 kinase activation downstream of TNFR1 signaling led to rapid apoptosis and necroptosis in microglia. Although all CNS cells appear to express RIPK1, RIPK3, and MLKL (Ofengeim et al., 2015), differences in expression levels and sensitivity thresholds among cell types may explain the variable susceptibility to death. We observed deleterious non-cell-autonomous effects when OPCs were co-cultured with mixed glial cells. Reduced myelination upon RIPK1 inhibition when OPCs were co-cultured with microglia or astrocytes is consistent with the dual deleterious and regenerative potential of inflammatory signaling, indicating basal RIPK1 activity may support secretion of OPC differentiation factors. However, in a more biologically relevant mixed glial culture, excess RIPK1 activation has detrimental effects on OPC differentiation and myelinating capacity. These findings suggest RIPK1 kinase activation may drive a neuroinflammatory CNS milieu that is harmful to oligodendrocyte function and survival, contributing to demyelination and MS pathology.

Because RIPK1 kinase activity can mediate inflammatory signaling and cell death (Najjar et al., 2016; Yuan et al., 2019; Zhu et al., 2018), the lack of death in astrocytes does not mean RIPK1 is not activated and potentially detrimental. We found astrocytes and microglia can strongly activate RIPK1, leading to an inflammatory transcriptional signature *in vitro*. In line with previous studies, some of the top transcription factors regulating these genes involve members of the NF-κB and AP-1 families (Najjar et al., 2016; Ofengeim et al., 2017; Zhu et al., 2018). Thus, RIPK1 kinase activation regulates a core transcriptional signature in various cell types. Neurons have been shown to regulate Toll-like receptor (TLR)- and viral-induced chemokine expression in a RIPK1-kinase-dependent manner independently of cell death (Daniels et al., 2017). Here, we identify astrocytes as unique CNS cells in which RIPK1 kinase activation regulates a death-independent inflammatory gene signature and microglia as cells in which RIPK1 regulates inflammation and cell death, with both processes appearing to occur concurrently. Previous studies have presented various mechanisms to explain the significance of this co-regulation, including a switch from cell death to inflammation in an IFNβ-dependent manner and persistent translation after cell lysis (Muendlein et al., 2020; Orozco et al., 2019). Crucially, we show that in the absence of cell death in MLKL-deficient astrocytes and microglia, RIPK1 activation mediates an inflammatory transcriptional program. We confirmed our findings in human cells, demonstrating RIPK1 kinase activation drives inflammatory gene transcription and necroptosis in iPSC-derived microglia but an interferon and antiviral gene response in necroptosis-resistant astrocytes. Thus, the ability of RIPK1 to propagate inflammation may be a critical function of RIPK1-expressing cells, irrespective of their ability to undergo RIPK1-kinase-mediated death.

Our study provides a comprehensive overview of the RIPK1-dependent transcriptional landscape in microglia and astrocytes. Consistent with the translational relevance of microglial RIPK1 kinase activation, the core RIPK1-kinase-dependent transcriptional signature contained many pro-inflammatory cytokines and chemokines, such as *Ccl3, Ccl4, Il1β, Il6*, and *Cxcl1*, elevated in MS patients and implicated in MS pathogenesis (Göbel et al., 2018; Khaibullin et al., 2017; Soleimani et al., 2019; Stampanoni Bassi et al., 2018). Importantly, we found a similar RIPK1-kinase-dependent gene signature in astrocytes. Our global transcriptomic approach confirmed the role of RIPK1 in modulating the lipid metabolism regulator *Ch25h* (Ofengeim et al., 2015), and CH25H deficiency is protective in EAE (Chalmin et al., 2015). We found RIPK1 kinase activation in microglia and astrocytes induced expression of various genes whose deficiency is protective in EAE or whose expression is increased in MS patients, including *Bhlhe40, Mir155hg, Rgs1*, and *Lcn2* (Al Nimer et al., 2016; Lee and Bou Dagher, 2016; Lin et al., 2014; Murugaiyan et al., 2011; O’Connell et al., 2010). In addition, lipidomic analysis of sterol intermediates demonstrated RIPK1 activation in astrocytes disrupts the cholesterol biosynthesis pathway. Thus, RIPK1 kinase activation in microglia and astrocytes drives a disease-relevant inflammatory gene expression profile and may regulate lipid- and metabolism-related genes implicated in MS pathogenesis.

We used two mouse strains to demonstrate the importance of RIPK1 kinase signaling in the EAE model of MS. Our data revealed RIPK1 inhibition can block increases in plasma neurofilament levels, suggesting an attenuation of neurodegeneration, and demonstrated that therapeutic administration of a CNS-penetrant RIPK1 kinase inhibitor effectively blocks disease symptoms. Furthermore, we provided evidence for the role of RIPK1 kinase signaling in the progressive NOD-EAE model, in which we observed spinal cord increases in RIPK1 activation, similar to our findings in progressive MS patients. Therapeutic RIPK1 kinase inhibition significantly attenuated disease progression in the NOD-EAE model. We confirmed the relevance of RIPK1-kinase-mediated signaling in microglia and astrocytes *in vivo* during EAE by RNA-seq. Although our *in vitro* analysis in response to acute RIPK1 kinase activation demonstrated a pro-inflammatory transcriptional signature, the *in vivo* EAE signature revealed specific and distinct RIPK1-kinase-dependent regulation in microglia and astrocytes. Homeostatic microglial markers and mitochondrial dysfunction pathways were modulated in a RIPK1-kinase-dependent manner. Although transcriptional regulation of inflammation does not appear to be a primary function of RIPK1 during EAE, the critical chemokine CCL2 was downregulated by RIPK1 kinase inhibition in astrocytes (Dogan et al., 2008; Kim et al., 2014; Van Der Voorn et al., 1999). We confirmed astrocytes downregulate cholesterol synthesis genes during EAE (Itoh et al., 2018) and demonstrated RIPK1-kinase-mediated signaling regulates ribosomal and cholesterol biosynthesis genes. Further study is needed to explore the role of RIPK1 in lipid metabolism, but our results of RIPK1 kinase activation modulating cholesterol biosynthesis in astrocytes *in vitro* and in EAE suggest this regulation may occur in various diseases. Thus, our study implicates RIPK1-kinase-dependent signaling in both astrocytes and microglia in neuroinflammation and MS pathology and suggests RIPK1 as a therapeutic target, particularly in progressive MS.

## STAR★METHODS

### RESOURCE AVAILABILITY

#### Lead contact

Further information and requests for resources and reagents should be directed to and will be fulfilled by the Lead Contact, Dimitry Ofengeim (dimitry.ofengeim@sanofi.com).

#### Materials availability

This study did not generate new unique reagents.

#### Data and code availability

The accession numbers for the RNA-seq data reported in this paper are GEO: GSE154228 (EAE astrocytes and microglia), GEO: GSE154230 (murine microglia and astrocytes *in vitro)* and GEO: GSE172027 (human astrocytes).

### EXPERIMENTAL MODEL AND SUBJECT DETAILS

#### Cell culture and plating

For murine mixed glial cultures, brains from four-day old C57BL/6 mice were harvested and pooled in DMEM/F12-Glutamax media with 10% FBS transferred into warm 0.25% trypsin (2mL/brain) and incubated at 37°C while rotating for 30 minutes. The tissues were dissociated and filtered through a 70μM cell strainer. The single-cell suspension was plated in T150 tissue culture flasks (1 flask/mouse) and the cells were fed with a complete medium change 5, 8 and 11 days later. Mixed glia were also seeded at 2.5x10^4 cells/well in PDL-coated black 96-well plates, 1.5x10^5 cells/well in 12-well plates, or 3x10^5 cells/well in 6-well plates and cultured for 12 days for use in *in vitro* experiments. On day 12 mixed glial cells were trypsinized and the single cell suspensions were counted. To isolate primary murine microglia, the EasySep Mouse CD11b Positive Selection Kit (StemCell #18970) was used with EasySep Magnets (StemCell #18000) according to manufacturer’s instructions. Primary murine astrocytes were isolated from the microglia negative fraction, with the EasySep Mouse APC Positive Selection Kit (StemCell #18452) and plated in DMEM high glucose with 10% FBS and N2 supplement (1:100 dilution; Thermo Fisher Scientific #17502-048). For cell viability, ELISA and MSD experiments, isolated microglia and astrocytes were plated at 5x10^4 cells/well in PDL-coated 96-well plates. For qRT-PCR experiments, microglia were seeded at 1.5x10^6 cells/well and astrocytes plated at 1x10^6 cells/well in 12-well plates. For western blot experiments, microglia were seeded at 3x10^6 cells/well and astrocytes at 2x10^6 cells/well in 6-well plates. Cells were typically rested for 24-48 hours prior to stimulation. Cortical neurons were isolated from E16.5 C57BL/6 embryos and seeded at 4x10^4 cells/well in PDL and laminin-coated 96-well plates. Neurons were maintained in Neurobasal media with L-glutamine and B27 supplement prior to treating at day 6 in culture. OPCs were isolated from 2-5 day old Sprague Dawley rat pups with anti-A2B5 microbeads (Miltenyi #130-093-388) and maintained in DMEM/F12 media with B27 supplement (GIBCO #12587010) and supplemented with bFGF (2ng/mL; Thermo Fisher Scientific #PHG0024) and PDGF-AA (2ng/mL; Thermo Fisher Scientific #PHG0035). OPCs were seed on top of mixed glia or directly into PDL-coated black 96-well plates at 1x10^4 cells/well. Human macrophages were differentiated from CD14+ monocytes isolated from donor PBMCs and maintained in RPMI with 10% FBS and hM-CSF (100ng/mL; R&D Systems #216-MC). Human iPSC-derived microglia (FujiFilm #01279) were seeded at 2x10^4 cells/well in PDL-coated 96-well plates or 2.5x10^5 cells/well in PDL-coated 12-well plates and maintained in iCell Glial Base Medium with supplements. Human astrocytes (GIBCO #N7805-100) were seeded at 2.5x10^4 or 5x10^5 cells/well on Matrigel-coated 96- or 12-well plates, respectively, and maintained in DMEM with 10% FBS and N2 supplement.

#### C57BL/6 and NOD-EAE induction and scoring

All animal studies were conducted in compliance with the ethical regulations and full approval of Sanofi’s Institutional Animal Care and Use Committee (IACUC) and Tufts University IACUC. Female C57BL/6J mice, at 8-10 weeks of age, were immunized with an emulsion of MOG_35-55_ peptide (250μg/mouse; New England Peptides) in 2mg/mL complete Freund’s adjuvant (CFA; Chondrex Inc #7009). The emulsion was delivered by two subcutaneous injections to the lower back in a volume of 100μL per injection site. *Bordetella pertussis toxin* (PTX; Sigma-Aldrich #P7208) was administered via tail vein injection on Day 0 and Day 2 at a dose of 280ng/animal in 100μL of PBS. Following EAE induction, mice were monitored daily for paralytic symptoms and scored for their clinical presentation using a progressive scoring system (Score 0: no disease; Score 1: flaccid tail; Score 2: hindlimb weakness; Score 3: hindlimb paralysis; Score 4: Front limb weakness or partial paralysis; Score 5: death). On days 11 to 13 mice were randomized into treatment groups upon reaching a clinical score of 1, and twice daily dosing (BID) of vehicle (0.5% methyl cellulose in water) or RIPK1_inh_-1 (30mg/kg and 60mg/kg) was initiated.

Female NOD/ShiLtJ mice, at 8-10 weeks of age, were immunized with an emulsion of MOG_35-55_ peptide (150μg/mouse) in CFA. The emulsion was delivered by two subcutaneous injections to the lower back in a volume of 100μL per injection site. PTX was administered via intraperitoneal injection on Day 0 and Day 2 (150ng/mouse in 200μL PBS). Following EAE induction, the mice were monitored daily for paralytic symptoms and scored for their clinical presentation. On day 28 the mice were randomized into treatment groups and BID dosing of vehicle (0.5% methyl cellulose in water) or RIPK1_inh_-2 (30mg/kg) was initiated.

#### Human brain processing and sample preparation

Frozen human brain tissue was obtained from the Human Brain and Spinal Fluid Resource Center at University of California Los Angeles (http://brainbank.ucla.edu). Post-mortem derived tissue (50mg) was homogenized and lysed in 1% Triton lysis buffer (TBS), samples were centrifuged (13000xg), and the supernatant was collected as the soluble fraction. Lysis buffers contained protease and phosphatase inhibitors (1:100 dilution; Thermo Fisher Scientific #78444). The pellet was further lysed with RIPA buffer and sonicated with a fine tip sonicator. Prior to loading on a gel for western blot analysis, samples were diluted in 6M urea (Millipore Sigma #108487) in loading buffer.

### METHOD DETAILS

#### Reagents

Reagents and their final concentrations were as follows: recombinant human TNF (50ng/mL; R&D Systems #210-TA), FLAG-Tagged human TNF (1μg/mL; Enzo ALX-522-008-C050), recombinant mouse TNF (50ng/mL; R&D Systems #410-MT), Necrostatin-1 s (30μM; EMD Millipore #504297), LCL161 Smac mimetic (1μM; Selleck Chem #S7009), caspase inhibitor zVAD-fmk (20μM; Enzo Life Sciences #ALX-260-020), TAK1 inhibitor 5Z-7-Oxozeaenol (100nM; Sigma-Aldrich #O9890), RIPK3 inhibitor GSK’872 (3μM;Tocris Bioscience #6492), IKK inhibitor TPCA-1 (5μM; Selleck Chem S2824), DRAQ7 (1μM; abcam ab109202), CellTracker Green CMFDA dye (20μM; Thermo Fisher Scientific #C7025).

#### Cell stimulation and viability

Primary cells were stimulated for 2 hours for MSD experiments, for 4 hours for qRT-PCR experiments, and for 20 hours for cell viability and protein secretion readouts by CellTiter-Glo and ELISA, respectively. For OPC-related conditioned media cells were treated in serum-free OPC media for 24 hours and conditioned media was applied to OPCs for 72 hours. Typically, primary cells were pre-treated with TAK1 (5z-7, 100nM), cIAP1/2 (Smac mimetic, 1 μM), pan-caspase (zVAD, 20 μM), and RIPK1 kinase inhibitors (Nec-1 s, 30 μM)for 1 hour prior to stimulation with recombinant murine or human TNF (50ng/mL). After 20 hours, cell viability was assessed via CellTiter-Glo 2.0 (Promega #G9243; according to manufacturer’s instructions) and normalized to DMSO.

#### Immunoblotting

Cells were lysed in RIPA buffer, with protease/phosphatase inhibitors, and sonicated to extract protein. Protein concentration was measured using the BCA reagent (Thermo Fisher Scientific) and protein samples were run on NUPAGE 4%–12% Bis-Tris Midi Gels (Life Technologies, NP0321) along with 10 μL of Chameleon Duo Pre-stained protein ladder (LI-COR, 928-60000). The protein was transferred to a nitrocellulose membrane using the iBlot Transfer machine and iBlot gel transfer Stacks PVDF Regular (Life Technologies, 1340109). The membranes were blocked in Odyssey TBS blocking buffer (LI-COR, 927-50000) containing 0.1% Tween 20 for 1 hour at room temperature (RT). Membranes were incubated overnight at 4°C with the following primary antibodies: pRIPK1 (phospho-S166; 1:1000; Cell Signaling #31122 and #65746), RIPK1 (1:1000; BD Biosciences #610459 and Cell Signaling #3493), pMLKL (phospho-S345; 1:1000; abcam #ab196436 and phospho-S358; 1:1000; Cell Signaling #91689), MLKL (1:1000; Cell Signaling #14993 and #37705), MBP (1:1000; abcam ab62631), caspase-8 (1:1000; Cell Signaling #9746), and β-actin (1:5,000; Sigma-Aldrich #A5441). Membranes were incubated with LI-COR secondary antibodies at a 1:5000 dilution, imaged on the LI-COR Odyssey CLx and quantified with FIJI.

#### Co-immunoprecipitation

Co-immunoprecipitation was performed in primary murine astrocytes, plating 5x10^6 cells per 15cm dish and culturing for 3 days. Astrocytes were treated for 0, 5 or 15 minutes with 1μg/mL FLAG-hTNF. Cells were lysed in Pierce IP lysis buffer, supplemented with protease/phosphatase inhibitors and N-Ethylmaleimide (10mM; Thermo Fisher Scientific #23030), and 1mg lysates were incubated with 100uL bead slurry of Pierce Anti-DYKDDDDK Magnetic Agarose Beads (Thermo Fisher Scientific #A36797) for 4 hours, rotating at 4°C. Samples were washed 3 times using 1mL lysis buffer and bound proteins were eluted using 50μL of 1X Laemmli Sample Buffer (Boston BioProducts #BP-111R) and boiling the samples at 95°C for 5 min. Beads were retrieved using magnetic stand and solubilized proteins were analyzed by immunoblotting.

#### Immunohistochemistry and RNAscope hybridization

Fresh frozen human brain tissue was sectioned at 10 μm with a Microm HM560 cryostat and slides were stored at −80°C until use. *In situ* hybridization and immunohistochemistry were performed with the Roche Ventana Medical Systems DISCOVERY ULTRA platform. Briefly, slides were thawed for 2 minutes and placed in 10% neutral buffered formalin for 24 hours. Slides were subsequently rinsed twice in PBS for 5 minutes and wet loaded into the Ventana. Sections were further permeabilized with cell conditioning for 8 minutes followed by target retrieval at 97°C and protease treatment (16 min at 37°C) (ACD mRNA dewax, #323222; ACD mRNA target retrieval VS universal, #323221; ACD mRNA protease, #322218). RNAscope 2.5 VS Probe-*Hs-RIPK1* (ACDBio #596589) was then hybridized for 2 h at 43°C followed by RNAscope amplification. Final signal detection was carried out using mRNA HRP (ACD RNAscope VS Universal HRP Detection Reagents #323210). Slides were then rinsed in dish soap and running water before another wet load in Ventana for subsequent immunohistochemistry procedure. Primary antibodies rabbit anti-IBA1 (1:500; Wako, #019-19741) or mouse anti-GFAP (1:10,000; BioLegend #837504, clone SMI 25) were diluted in antibody dilution buffer (Roche, #ADB250) and applied to the slides (100 μL/slide). Slides were incubated at 37°C for 1 hour prior to addition of UltraMap anti-Rabbit HRP (Roche, #760-4315) or anti-Mouse HRP (Roche, #760-4313). The signal detection was carried out using Discovery Purple (Roche, #760-229) and counterstaining with hematoxylin (Roche, #760-2021) and bluing solution (Roche, #760-2037). Finally, slides were removed from the Ventana, dehydrated, and coverslipped with Cytoseal XYL. Slides were dried and then imaged using a Zeiss Imager.Z1 microscope equipped with a Zeiss AxioCam MRm digital camera and Zen Pro 2012 Imaging Software. HALO (Indica Labs, version 2.3) was used to assess RIPK1 ISH and IBA1+ or GFAP+ marker co-localization.

#### Immunocytochemistry and immunofluorescence

Cells were fixed with 4% PFA for 20 minutes at RT prior to washing 3 times for 5 minutes in 0.2% PBT (0.2% Triton X-100 in PBS) and blocking with 10% donkey serum/0.2% PBT for 1 hour. Cells were incubated in the primary antibodies CSF1R (1:100; R&D Systems #AF3818), MBP (1:500; Biorad #MCA409S) and Olig2 (1:500; EMD Millipore #AB9610) diluted in 5% donkey serum/0.2% PBT overnight at 4°C. The next day, cells were washed and incubated in secondary antibodies from Life Technologies at a 1:1000 dilution, diluted in 5% donkey serum/0.2% PBT for 1 hour. Cells were then incubated in DAPI (1:20,000 in PBS) for 5 minutes, washed, and rinsed in PBS. Plates were imaged on the Phenix Opera or the IN Cell Analyzer 2200 with 9 fields of view acquired per well (Phenix: 0.384mm^2^ and IN Cell: 0.443mm^2^ per field of view), with all treatments done in at least technical triplicate per experiment. Quantification was performed on Harmony software (version 4.9) or the IN Cell Developer Toolbox (version 1.9.2), calculating the Olig2+ cell counts and the sum of the area (in μm^2^) of CSF1R+ and MBP+ staining. MBP+ area was normalized to Olig2 counts prior to normalization to DMSO control.

#### Incucyte live imaging

Cells were imaged in the Incucyte Zoom (Essen Bioscience). For monitoring dead cells, DRAQ7 was added at 1μM for 1 hour prior to stimulating cells in media containing 1μM DRAQ7. Microglia were labeled with CellTracker Green CMFDA by incubating in 20μM dye for 45 minutes at 37°C. Cells were washed once, centrifuged, and then added to mixed glial cultures at 4x10^4 cells/well prior to imaging in the Incucyte.

#### RIPK1 MSD ELISA

Lysates were prepared from cultured cells or frozen tissues homogenized in Cell Signaling lysis buffer (Cell Signaling #9803) or RIPA buffer, with protease/phosphatase inhibitors and benzonase (1:500 dilution; Sigma E1014). Biotinylated RIPK1 capture antibody (BD Bioscience #610459) was diluted in PBS to 1mg/mL and added to MSD Gold 96-well small spot streptavidin plates and incubated with shaking (700rpm) for 1 hour at RT. After blocking with MSD Blocker A for 2 hours with shaking (700rpm), 25-30 μL of lysates were incubated overnight at 4°C with shaking (600rpm). Phospho-RIPK1 (Cell Signaling #31122) or RIPK1 (Cell Signaling #3493) were diluted 1:500 in MSD Blocker A and added as detection antibodies for 1.5 hours with shaking (700rpm) at RT. Secondary detection antibody (goat anti-rabbit antibody, sulfo-TAG labeled, Meso Scale Discovery, R32AB-1) was diluted 1:1000 in MSD Blocker A and added for 1 hour at RT with shaking (700rpm). The assay was developed using 150 μL of 2x MSD read buffer (R92TC-3) and plates were read on an MSD Meso Sector imager S600. ECL counts were normalized to untreated controls or protein concentration.

#### LC-MS analysis of sterols

After thawing cell pellets at RT for 30 minutes, PBS (0.75mL) was added and mixed. The homogeneous solution was transferred to Screw Cap Culture Glass tubes (16x100mm). 3mL extraction solution (2:1 choloroform/methanol with 50ng/ml D5-zymosterol) was added using a glass pipette. The tube was capped and vortexed for 5 minutes. After centrifuging at 3,500 rpm for 5 minutes at 25°C, the lower layer organic solution was carefully transferred to a new glass tube with a glass pipette. 2mL of chloroform was added to the original tubes for a second extraction. The lower layer was combined into the tube containing the initial extraction. 2mL of PBS was added to the extracted organic solutions. After mixing and centrifuging at 3,500 rpm for 5 minutes, the lower layer was transferred into a new glass vial and dried under N2. The extracted lipids were reconstituted into 9:1 methanol:water (150uL) and transferred to a deactivated Qsert Vial for LC-MS analysis. Standard curves were prepared in 0.75mL PBS buffer. 14 sterol standards (linearity range 5-500 ng/mL) in the cholesterol biosynthesis pathway were prepared in a similar way. LC-MS was performed using a Waters ACQUITY UPLC system connected to a Sciex Q-TRAP 6500 mass spectrometer. Analyte (5 mL) was injected into the system with a partial loop in the needle overfill mode. An atmospheric pressure chemical ionization (APCI)+ source was used with the following parameters: curtain gas 16.0; ionSpray voltage 5.5 kV; temperature 380°C; ion source gas: 80 and 0; declustering potential 70V; entrance potential 10V; collision energy 38V; and collision cell exit potential 10V. The sterols were separated on a Waters ACQUITY UPLC CSH C18 Column (1.7 μm, 2.1 mm × 150 mm). The autosampler and column oven were maintained at 10°C and 30°C, respectively. The mobile phase consisted of Solvent A: 96% methanol with 0.1% acetic acid and Solvent B: methanol with 0.1% acetic acid. The gradient program was isocratic 100% A at 0.15 mL/min for 18 minutes, followed by a linear curve to 100% B at 18.5min, with the remainder of the run at 100% B at 0.25mL/min. 25 minutes per sample. MS Data were analyzed in MultiQuant. All data are normalized to the corresponding internal standard (D5-zymosterol), and data are plotted as concentration in nM per million cells.

#### RNA extraction, cDNA synthesis, and quantitative real-time PCR

RNA isolation from the brain was performed following manufacturer’s instructions with the RNeasy Lipid Tissue Mini Kit (QIAGEN #74804), with approximately 50 mg of human brain tissue homogenized in 1 mL of QIAzol Lysis Reagent with a handheld homogenizer. Cells were lysed in 350mL RLT Plus Lysis Buffer and RNA was extracted using the QIAGEN RNeasy Plus Mini kit (QIAGEN #74136) following manufacturer’s instructions. RNA quantity was measured with the Nanodrop 8000 and cDNA was synthesized using 500ng to 1mg total RNA with the Quantitect Reverse Transcription Kit (QIAGEN #205313) according to manufacturer’s instructions. For qRT-PCR, samples were run in a 384-well plate in triplicate on the QuantStudio 7 Real-Time PCR system (Applied Biosystems) with 2 mL cDNA per well used with Fastlane master mix, H_2_O and Taqman probes in a total volume of 20 μL. Relative gene expression was normalized to the housekeeping gene Rpl37a, and determined using the ΔΔCT method.

#### RNA sequencing and analysis

Primary murine microglia and astrocytes were stimulated for 4 hours as indicated, and cells were lysed in 350mL RLT Plus Lysis Buffer. RNA was extracted using the QIAGEN RNeasy Plus Mini kit following manufacturer’s instructions and RNA quantification and quality assessment was performed with the Agilent 2100 Bioanalzyer. The RNA was used for sequencing library preparation with polyA selection and sequenced on BGI DNBseq with paired-end 150bp reads. The samples’ raw RNA-seq data were uploaded, processed, and analyzed with the RNA-seq process pipeline in OmicSoft Array Studio software (version 10.1.1.3), including data quality control, alignment, quantification, normalization and log2 transformation to obtain the expression values (log2 FPKM) for each sample. The differentially expressed genes from each comparison were derived by paired t test on sample expressions with different cutoffs, e.g., ∣FC∣ >1.5 and p value < 0.05. ToppGene Suite (https://toppgene.cchmc.org/) and Ingenuity Pathway Analysis (QIAGEN) were used for gene ontology, pathway, and gene-disease association analysis.

#### siRNA silencing

Primary murine astrocytes were isolated and plated at 3x10^5 cells per well in a 24-well plate. 72 hours later, 1μM Accell Mouse Ripk1 siRNA SMARTpool (Dharmacon #E-040150-00-0010) was used to treat the astrocytes. After 72 hours, the cells were treated with T/5z-7 or T/S/Z for 4 hours as described previously and lysed for RNA extraction.

#### EAE Spinal Cord Isolation and FACS Sorting

EAE was induced in female C57BL/6J mice which were dosed for 3 days upon reaching a clinical score of 1, with BID dosing of vehicle (20% Captisol) or RIPK1_inh_-1 (50mg/kg). Mice were sacrificed and perfused with PBS. Spinal cords were extracted, chopped in Accutase (1mL/sample; Sigma), and homogenized with a 19-gauge needle. Samples were incubated at 4°C for a total of 20 minutes and homogenized with a 21-gauge needle at intervals of 10 minutes. Cells were then filtered through a 100μm cell strainer. Samples were pelleted, resuspended in 3mL PBS and 4mL 25% BSA in PBS, and centrifuged (1200 g, 10 minutes). Supernatants were aspirated to remove excess myelin and samples were resuspended in 5mL 25% BSA. The spin was repeated, and supernatants discarded. Cells were resuspended in 100μL FACS staining buffer (2% FBS, 2mM EDTA, 1% P/S in PBS) and treated with Fc block (CD16/32, 1:100; BD Biosciences, #553142) for 5 minutes. Cells were stained for FACS sorting at 4°C for 45 minutes with the following antibodies: anti-CD45 BUV395 (1:50; BD Biosciences #564279, clone 30-F11), anti-CD11b BV421 (1:50; BD Biosciences #562605, clone M1/70), anti-CX3CR1 BV711 (1:50; Biolegend, #149031, clone SA011F11), anti-CD140a PE (1:50; Biolegend, #135905, clone APA5), and anti-GLAST APC (1:10; Miltenyi Biotec, #130-095-822, clone ASCA-1). Astrocytes were defined as GLAST^+^CD11b^−^CD45^−^CD140a^−^ while microglia were defined as CD11b^+^CD45^int^CX3CR1^+^.

#### Plasma Neurofilament-H

Murine plasma neurofilament-heavy (NF-H) was assessed on 5μL murine plasma using the human SimplePlex Assay from R&D Systems (the kit is cross reactive with murine NF-H; Protein Simple #SPCKB-PB-000519) according to manufacturer’s instructions. The SimplePlex Assay performed on the Ella platform utilizes a microchip with capillaries coated with NF-H antibodies.

#### ELISA and Milliplex

Cell culture supernatants from astrocytes were subjected to murine IL-6 ELISA (R&D Systems) according to the manufacturer’s instructions. The concentration of cytokines in the media was adjusted based on the standard curve. Cell culture supernatants from microglia were run on a custom MILLIPLEX MAP mouse cytokine/chemokine magnetic bead panel (Millipore Sigma) following manufacturer’s instructions to determine the concentrations of CXCL1 and CXCL2.

### QUANTIFICATION AND STATISTICAL ANALYSIS

Data are presented as mean ± SD for *in vitro* experiments and mean ± SEM for *in vivo* and *ex vivo* samples. Statistical analyses were performed using GraphPad Prism software, version 8.4.2 and p values are indicated by * ≤ 0.05, ** ≥ 0.01, *** ≤ 0.001, and **** ≥ 0.0001. P values were determined by paired or unpaired 2-tailed Student’s t test with Welch’s correction, or one-way ANOVA or two-way ANOVA with post hoc Tukey, Sidak or Dunnett’s test. N represents biological replicates, with each point depicting an individual mouse or human sample, or the mean of technical replicates from cells isolated from separate litters of mice. When data are shown as representative of independent experiments, technical replicates are depicted from a representative experiment, and statistical analysis was not performed on graphs depicting technical replicates.

## Supplementary Material

Supplementary data

## Figures and Tables

**Table:** KEY RESOURCES TABLE

REAGENT or RESOURCE	SOURCE	IDENTIFIER
Antibodies		
Anti-p-S166 mRIPK1	Cell Signaling Technology	Cat# 31122; RRID:AB_2799000
Anti-p-S166 hRIPK1	Cell Signaling Technology	Cat# 65746; RRID:AB_2799693
Anti-RIPK1	BD Biosciences	Cat# 610459; RRID:AB_397832
Anti-RIPK1 (D94C12)	Cell Signaling Technology	Cat# 3493; RRID:AB_2305314
Anti-p-S345 mMLKL	Abcam	Cat# ab196436; RRID:AB_2687465
Anti-p-S358 hMLKL	Cell Signaling Technology	Cat# 91689; RRID:AB_2732034
Anti-MLKL (D216N)	Cell Signaling Technology	Cat# 14993; RRID:AB_2721822
Anti-MLKL (D6W1K)	Cell Signaling Technology	Cat# 37705; RRID:AB_2799118
Anti-Caspase-8 (1C12)	Cell Signaling Technology	Cat# 9746; RRID:AB_2275120
Anti-β-Actin	Sigma-Aldrich	Cat# A5441; RRID:AB_476744
Anti-MBP	Abcam	Cat# ab62631; RRID:AB_956157
Anti-MBP	Bio-Rad	Cat# MCA409S; RRID:AB_325004
Anti-Olig2	Millipore	Cat# AB9610; RRID:AB_570666
Anti-Iba1	Wako	Cat# 019-19741; RRID:AB_839504
Anti-GFAP	BioLegend	Cat# 837504; RRID:AB_2632641
Anti-CSF1R	R&D Systems	Cat# AF3818; RRID:AB_884158
CD45 (BUV395)	BD Biosciences	Cat# 564279; RRID:AB_2651134
CD11b (BV421)	BD Biosciences	Cat# 562605; RRID:AB_11152949
CX3CR1 (BV711)	BioLegend	Cat# 149031; RRID:AB_2565939
CD140a (PE)	BioLegend	Cat# 135905; RRID:AB_1953268
GLAST (APC)	Miltenyi Biotec	Cat# 130-095-822; RRID:AB_10829302
Goat anti-Rabbit Antibody, SULFO-TAG labeled	Meso Scale Discovery	Cat# 32AB
Donkey Anti-Rat IgG H&L (Alexa Fluor 594)	ThermoFisher	Cat# A-21209; RRID:AB_2535795
Donkey Anti-Sheep IgG H&L (Alexa Fluor 647)	ThermoFisher	Cat# A-21448; RRID:AB_2535865
Donkey Anti-Rabbit IgG H&L (Alexa Fluor 488)	ThermoFisher	Cat# A-21206; RRID:AB_2535792
IRDye® 800CW Donkey anti-Rabbit	LI-COR Biosciences	Cat# 926-32213; RRID:AB_2715510
IRDye® 800CW Donkey anti-Mouse	LI-COR Biosciences	Cat# 926-32212; RRID:AB_621847
IRDye® 680RD Donkey anti-Mouse	LI-COR Biosciences	Cat# 926-68072; RRID:AB_10953628
Biological samples		
Human post-mortem brain samples	Human Brain and Spinal Fluid Resource Center at University of California Los Angeles	http://brainbank.ucla.edu
Chemicals, peptides, and recombinant proteins		
Recombinant mouse TNF	R&D Systems	Cat# 410-MT
Necrostatin-1s	EMD Millipore	Cat# 504297
LCL161 Smac mimetic	Selleck Chem	Cat# S7009
zVAD-fmk	Enzo	Cat# ALX-260-020
5Z-7-Oxozeaenol	Sigma Aldrich	Cat# O9890
GSK’872	Tocris Bioscience	Cat# 6492
TPCA-1	Selleck Chem	Cat# S2824
DRAQ7	Abcam	Cat# ab109202
CellTracker Green CMFDA dye	Thermo Fisher Scientific	Cat# C7025
N2 supplement	Thermo Fisher Scientific	Cat# 17502-048
B27 supplement	GIBCO	Cat# 12587010
Human FGF-basic	Thermo Fisher Scientific	Cat# PHG0024
Human PDGF-AA	Thermo Fisher Scientific	Cat# PHG0035
Recombinant human M-CSF	R&D Systems	Cat# 216-MC
Recombinant human TNF	R&D Systems	Cat# 210-TA
FLAG-tagged human TNF	Enzo	Cat# ALX-522-008-C050
MOG_35-55_ peptide	New England Peptide	Sequence H2N-MEVGWYRSPFSRVVHLYRNGK-OH
Critical commercial assays		
anti-A2B5 microbeads	Miltenyi	Cat# 130-093-388
EasySep Mouse CD11b Positive Selection Kit	StemCell	Cat# 18970
CellTiter-Glo 2.0	Promega	Cat# G9243
RNeasy Lipid Tissue Mini Kit	QIAGEN	Cat# 74804
RNeasy Plus Mini kit	QIAGEN	Cat# 74136
Quantitect Reverse Transcription Kit	QIAGEN	Cat# 205313
EasySep Mouse APC Positive Selection Kit	StemCell	Cat# 18452
TaqMan Fast Advanced Master Mix	ThermoFisher	Cat# 4444557
Human Neurofilament-heavy SimplePlex Assay	R&D Systems	Protein Simple Cat# SPCKB-PB-000519
Mouse IL-6 Quantikine ELISA	R&D Systems	Cat# M6000B
Complete Freund’s adjuvant	Chondrex	Cat# 7009
*Bordetella pertussis* toxin	Sigma-Aldrich	Cat# P7208
Deposited data		
Mouse astrocyte RNA-seq	This paper	GEO: GSE154230
Human astrocyte RNA-seq	This paper	GEO: GSE172027
Mouse EAE astrocyte and microglia RNA-seq	This paper	GEO: GSE154228
Mouse microglia RNA-seq	This paper	GEO: GSE154230
Experimental models: Cell lines		
Human astrocytes	GIBCO	Cat# N7805-100
Human iPSC microglia	FujiFilm	Cat# 01279
Experimental models: Organisms/strains		
NOD/ShiLtJ mice	The Jackson Laboratory	Cat# JAX:001976; RRID:IMSR_JAX:001976
MLKL-KO mice	Provided by Dr. Warren Alexander	N/A
C57BL/6J mice	The Jackson Laboratory	Cat# JAX:000664; RRID:IMSR_JAX:000664
Oligonucleotides		
Accell Mouse Ripk1 siRNA SMARTpool	Dharmacon	Cat# E-040150-00-0010
RNAscope Probe - HuRipk1	Advanced Cell Diagnostics	Cat# 596589
RPL37A-VIC: Hs01102345 (TaqMan Gene Expression Assays)	ThermoFisher	N/A
RIPK1-FAM: Hs01041869 (TaqMan Gene Expression Assays)	ThermoFisher	N/A
CXCL2-FAM: Hs00601975 (TaqMan Gene Expression Assays)	ThermoFisher	N/A
IL6-FAM: Hs00174131 (TaqMan Gene	ThermoFisher	N/A
Ripk1-FAM: Mm00436354 (TaqMan Gene Expression Assays)	ThermoFisher	N/A
Cxcl1-FAM: Mm04207460 (TaqMan Gene Expression Assays)	ThermoFisher	N/A
Cxcl2-FAM: Mm00436450 (TaqMan Gene Expression Assays)	ThermoFisher	N/A
Csf2-FAM: Mm01290062 (TaqMan Gene Expression Assays)	ThermoFisher	N/A
Il6-FAM: Mm00446190 (TaqMan Gene Expression Assays)	ThermoFisher	N/A
Software and algorithms		
Incucyte Zoom	Essen Bioscience	Zoom 2018A; RRID:SCR_019874
Harmony	PerkinElmer	Version 4.9
IN Cell Developer Toolbox	GE Healthcare	Version 1.9.2
Prism	GraphPad	Version 8.4.2; RRID:SCR_002798
MultiQuant	SCIEX	Version 3.0
OmicSoft Array Studio	QIAGEN	Version 10.1.1.3
ZEN Pro 2012	ZEISS	RRID:SCR_013672
HALO	Indica Labs	Version 2.3; RRID:SCR_018350
ImageJ	National Institutes of Health	RRID:SCR_003070
Discovery Workbench	Meso Scale Discovery	Version 4.0; RRID:SCR_019192
LI-COR Image Studio Software	LI-COR Biosciences	Version 5.2; RRID:SCR_015795

## Highlights

- Insoluble RIPK1 activity and expression are elevated in progressive forms of MS
- RIPK1 activation in microglia and astrocytes drives an inflammatory gene signature
- Therapeutic RIPK1 inhibition attenuates symptoms in a progressive mouse model of MS
