# Supplementary material for: RIPK1 activation mediates neuroinflammation and disease progression in multiple sclerosis
Source: Cell Rep. Author manuscript; Available in PMC 2022 Mar 12. (PMC8917516; doi:10.1016/j.celrep.2021.109112)
Supplement: Supplementary data [file NIHMS1783515-supplement-Supplementary_data.pdf]

Cell Reports, Volume *35*

Supplemental information

RIPK1 activation mediates neuroinflammation and disease progression in multiple sclerosis

Matija Zelic, Fabrizio Pontarelli, Lisa Woodworth, Cheng Zhu, Amy Mahan, Yi Ren, Michael LaMorte, Ross Gruber, Aislinn Keane, Pequita Loring, Lilu Guo, Tai-he Xia, Boyao Zhang, Pontus Orning, Egil Lien, Alexei Degterev, Timothy Hammond, and Dimitry Ofengeim

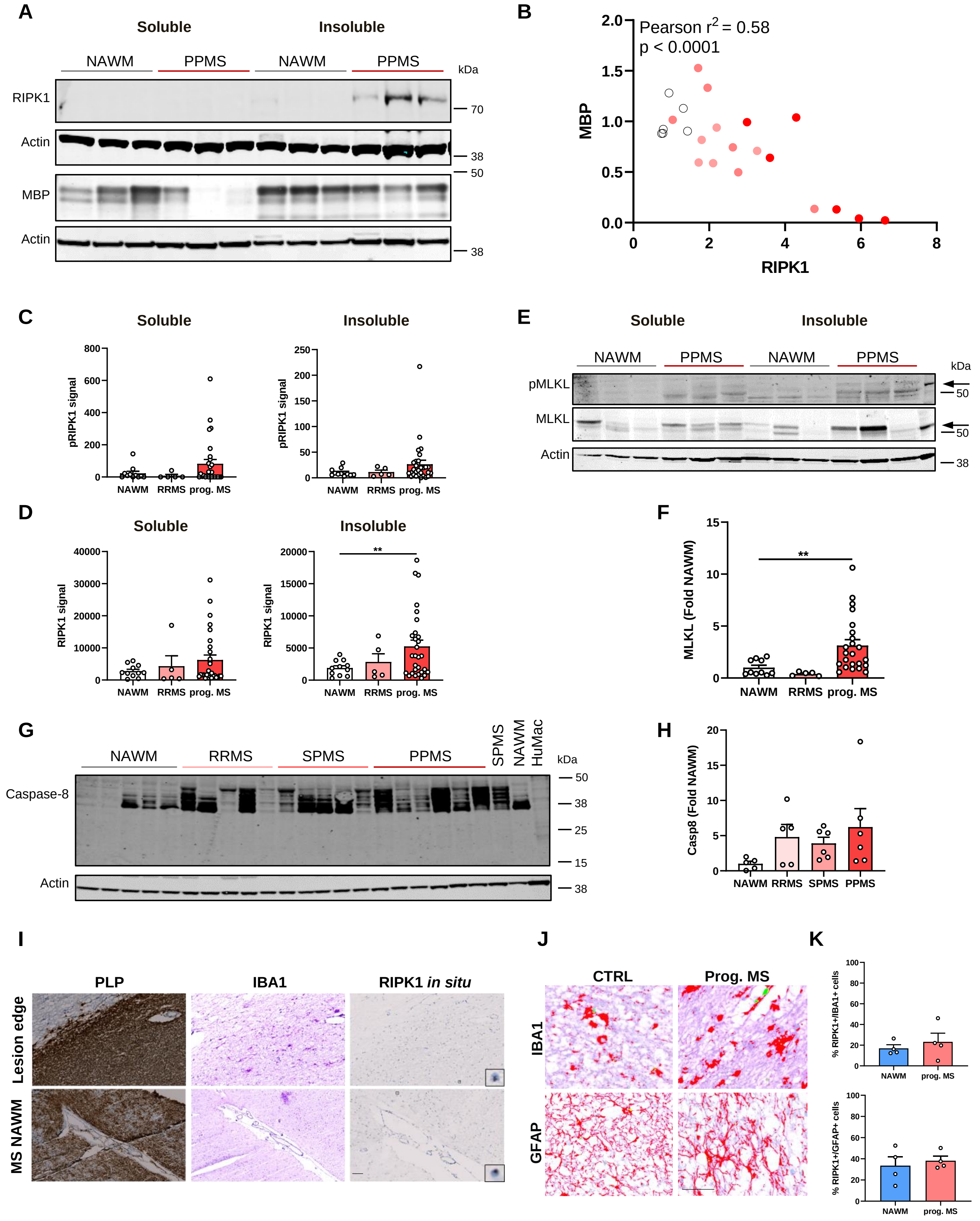
A
Soluble
Insoluble
NAWM
PPMS
NAWM
PPMS
kDa
RIPK1
Actin
MBP
Actin
70
38
50
38
B
Pearson r^2 = 0.58
$p < 0.0001$
MBP
RIPK1
C
Soluble
Insoluble
pRIPK1 signal
NAWM
RRMS
prog. MS
D
Soluble
Insoluble
RIPK1 signal
**
NAWM
RRMS
prog. MS
E
Soluble
Insoluble
NAWM
PPMS
NAWM
PPMS
kDa
pMLKL
MLKL
Actin
50
50
38
F
MLKL (Fold NAWM)
**
NAWM
RRMS
prog. MS
G
NAWM
RRMS
SPMS
PPMS
SPMS
NAWM
HuMac
kDa
Caspase-8
Actin
50
38
25
15
38
H
Casp8 (Fold NAWM)
NAWM
RRMS
SPMS
PPMS
I
PLP
IBA1
RIPK1 *in situ*
Lesion edge
MS NAWM
J
CTRL
Prog. MS
IBA1
GFAP
K
% RIPK1+/IBA1+ cells
NAWM
prog. MS
% RIPK1+/GFAP+ cells
NAWM
prog. MS

Figure S1: RIPK1 is elevated in progressive MS, Related to **Figure 1**
(A) Protein expression assessed by immunoblotting soluble (TBS/Triton) and insoluble (RIPA/urea) brain lysates from NAWM control (sample ID 1, 3 and 5) and PPMS (sample ID 16, 19 and 21) patients (n=3 per group) probed with antibodies against RIPK1, MBP and β-actin.
(B) Correlation of soluble MBP to insoluble RIPK1 levels assessed by immunoblotting, normalized to NAWM control (n=5 RRMS and n=6 NAWM, SPMS and PPMS samples).
(C, D) MSD assay quantifying pRIPK1 **(C)** and total RIPK1 **(D)** levels in soluble and insoluble brain lysates from NAWM control, RRMS and progressive MS (prog. MS) samples (n=11 NAWM, n=5 RRMS and n=29 progressive MS samples).
(E) Protein expression assessed as in **(A)** for pMLKL, MLKL and β-actin. Positive control is lysate from T/S/Z-treated human macrophages, as indicated by arrow.
(F) Quantification of MLKL expression in insoluble protein fraction from control and MS patient samples, normalized to NAWM control (n=10 NAWM, n=5 RRMS and n=23 prog. MS samples).
(G) Protein expression of soluble brain lysates from control and MS patients probed with antibodies against Caspase-8 and β-actin. Human macrophage lysate is included as a control.
(H) Quantification of blots in **(G)** normalized to NAWM control (n=5 NAWM and RRMS, n=6 SPMS and PPMS samples).
(I) PLP and IBA1 immunohistochemistry (IHC) and RIPK1 *in situ* hybridization (ISH) staining of normal appearing white matter (NAWM) and lesion edge areas of progressive MS brain tissue. Inset depicts RIPK1+ ISH as brown dots. Scale bar: 100 µM.
(J) Dual RIPK1 ISH and IBA1/GFAP IHC staining of control and progressive MS brain tissue. Digital co-localization of RIPK1 and IBA1 or GFAP is depicted by pseudo-yellow color while RIPK1+ ISH appears green and IBA1+ or GFAP+ IHC appears red. Images are representative of 4 NAWM and 4 progressive MS samples. Scale bar: 50 µM.
(K) Quantification of co-localized RIPK1+/IBA1+ or RIPK1+/GFAP+ cells (n=4 per group). 2-3 areas of similar size were quantified per sample, including lesion area in progressive MS samples.
Error bars represent mean ± SEM. One-way ANOVA with Dunnett post-hoc test **(D and F)** was performed. ** $p<0.01$. T: TNF, S: Smac, Z: zVAD.

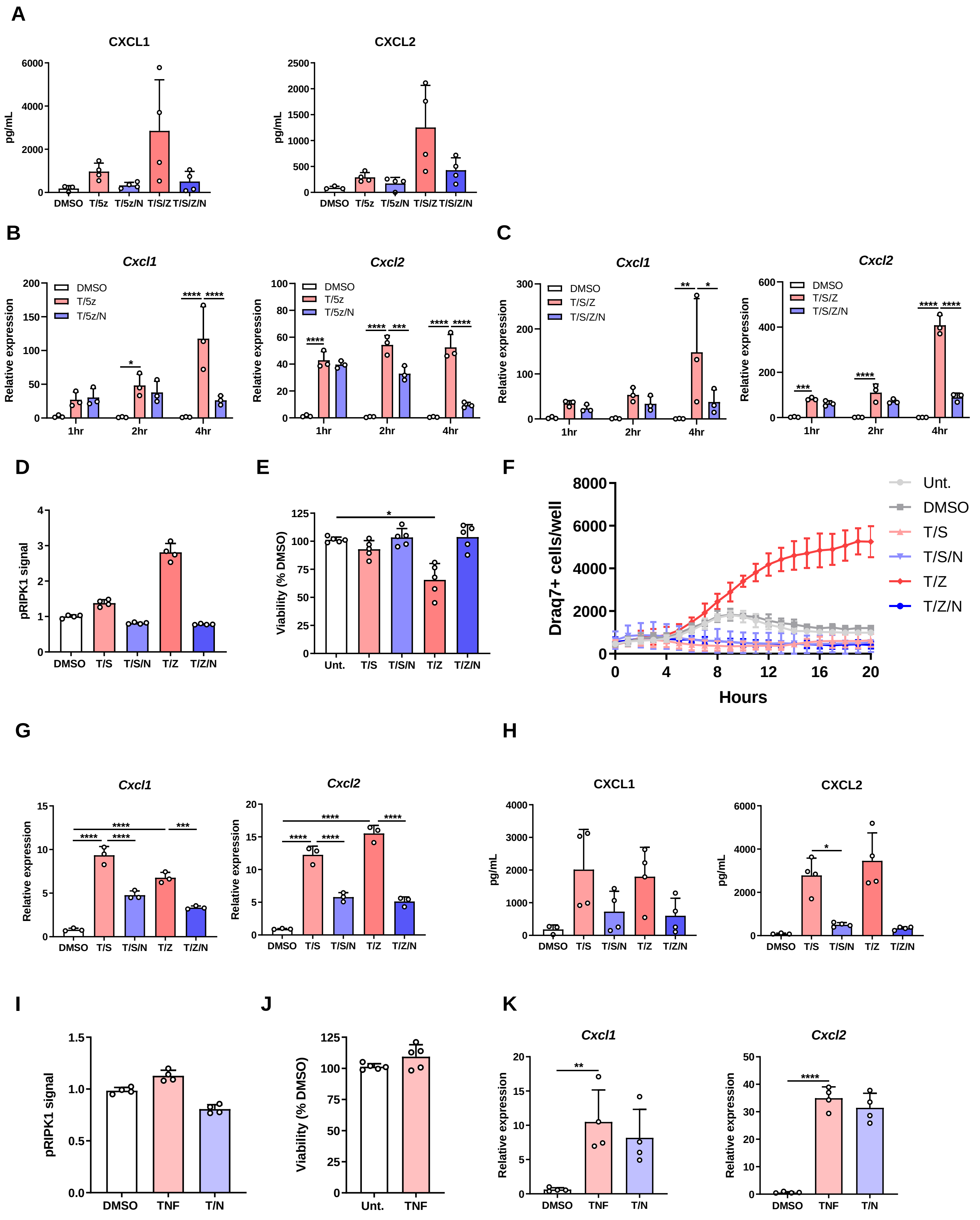
A
CXCL1
pg/mL
DMSO T/5z T/5z/N T/S/Z T/S/Z/N
CXCL2
B
Cxcl1
Relative expression
DMSO
T/5z
T/5z/N
1hr 2hr 4hr
Cxcl2
C
Cxcl1
DMSO
T/S/Z
T/S/Z/N
Cxcl2
D
pRIPK1 signal
DMSO T/S T/S/N T/Z T/Z/N
E
Viability (% DMSO)
Unt. T/S T/S/N T/Z T/Z/N
F
Draq7+ cells/well
Hours
Unt.
DMSO
T/S
T/S/N
T/Z
T/Z/N
G
Cxcl1
Cxcl2
H
CXCL1
CXCL2
I
pRIPK1 signal
DMSO TNF T/N
J
Viability (% DMSO)
Unt. TNF
K
Cxcl1
Cxcl2
DMSO TNF T/N

Figure S2. RIPK1 kinase activation regulates pro-inflammatory signaling and cell death in murine microglia *in vitro* but is dispensable for TNF-induced pro-inflammatory signaling, Related to **Figure 2**

(A) Quantification of CXCL1 and CXCL2 levels by multiplex immunoassay in microglia treated for 20h (n=3 for DMSO, n=4 for other stimulations).

(B, C) Relative gene expression normalized to *Rpl37a* in microglia treated for 1h, 2h or 4h with T/5z **(B)** or T/S/Z **(C)** with or without Nec-1s (n=3).

(D) MSD assay quantifying pRIPK1 levels in microglia treated for 2h.

(E) Viability of microglia stimulated for 20h (n=5).

(F) Quantificatoin of DRAQ7 microglia imaged in the IncuCyte ZOOM.

(G) Relative gene expression in microglia treated for 4h (n=3).

(H) Quantification of CXCL1 and CXCL2 levels by multiplex immunoassay in microglia treated for 20h (n=3 for DMSO, n=4 for other stimulations).

(I) MSD assay quantifying pRIPK1 levels in microglia treated for 2h.

(J) Primary microglia were stimulated with TNF for 20h and cell viability was measured using CellTiter-Glo assay (n=5).

(K) Relative gene expression in microglia treated for 4h (n=4).

Error bars represent mean ± SD. Data depict technical replicates and are representative of 2 **(D and I)** or 3 **(F)** independent experiments. One-way **(E, G, H and K)** or two-way ANOVA **(B and C)** with Tukey post-hoc test was performed. * $p<0.05$, ** $p<0.01$, *** $p<0.001$, **** $p<0.0001$. T: TNF, S: Smac, Z: zVAD, N: Nec-1s.

A

RDA TFs

Transcription Factor	pValue
**NFKBIA**	2.1E-39
**STAT3**	4.55E-29
**RELA**	1.82E-27
**TP53**	2.14E-25
**NFKB1**	3.24E-24
**EGR1**	9.13E-24
**JUN**	5.34E-23
**NFATC2**	5.47E-23
TP73	1.06E-19
STAT1	1.26E-19
**SP1**	4.21E-19
NFAT5	2.52E-18
**CEBPB**	3.65E-18
ZFP36	3.48E-17
**MYB**	4.71E-17
**FOSL1**	1.06E-16
HMGB1	1.12E-16
**HIF1A**	1.17E-16
IRF3	3.02E-16
MEOX2	2.9E-15

B

Necroptosis TFs

Transcription Factor	pValue
**NFKBIA**	4.05E-41
**JUN**	8.36E-35
**TP53**	1.95E-34
**HIF1A**	4.34E-30
**EGR1**	5.9E-30
**STAT3**	2.73E-28
**NFKB1**	2.24E-24
SMAD4	1.57E-22
**RELA**	1.83E-22
**SP1**	4.43E-21
FOXO3	1.89E-20
CREB1	1.93E-20
**FOSL1**	2.03E-19
**MYB**	3.15E-19
FOS	3.34E-19
**CEBPB**	3.51E-19
**NFATC2**	6.64E-19
SMAD3	6.8E-19
EP300	1.19E-18
FOXO1	3.03E-18

C

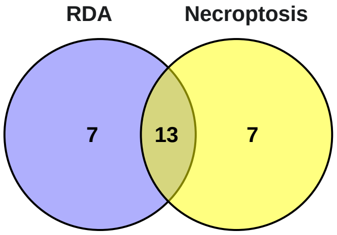

D

Rank	Biological Process	pValue
1	inflammatory response	1.70E-20
2	response to molecule of bacterial origin	1.02E-17
3	defense response	5.16E-17
4	cytokine-mediated signaling pathway	7.16E-17
5	response to other organism	7.77E-17
6	response to external biotic stimulus	8.05E-17
7	response to lipopolysaccharide	1.05E-16
8	response to biotic stimulus	1.29E-16
9	response to cytokine	2.23E-16
10	response to bacterium	3.30E-15

E

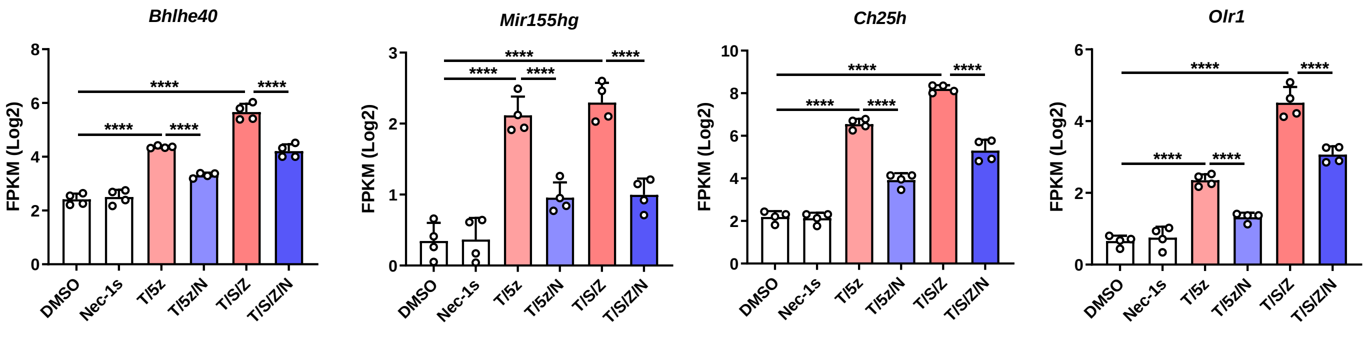

Figure S3. RIPK1 kinase activation regulates a pro-inflammatory gene signature in murine microglia *in vitro,* Related to **Figure 2**

(A, B) Top upstream transcriptional regulators predicted from Ingenuity Pathway Analysis (IPA) for RIPK1 kinase-dependent genes in microglia stimulated with T/5z **(A)** or T/S/Z **(B)** with cut-off |FC|>1.5, p<0.05. Overlapping transcription factors in bold.

(C) Overlap of predicted transcriptional regulators in **(A and B)**.

(D) Gene ontology terms for biological processes from the ToppGene Suite for the core microglial RIPK1 kinase-dependent gene list from the overlap of T/5z and T/S/Z stimulations (cut-off |FC|>2, p<0.05).

(E) Expression of individual microglial genes stimulated for 4h as assessed by RNA-seq (n=4).

Error bars represent mean ± SD. Paired Student's *t* test was performed. **** p<0.0001.

T: TNF, 5z: 5z-7, S: Smac, Z: zVAD, N: Nec-1s.

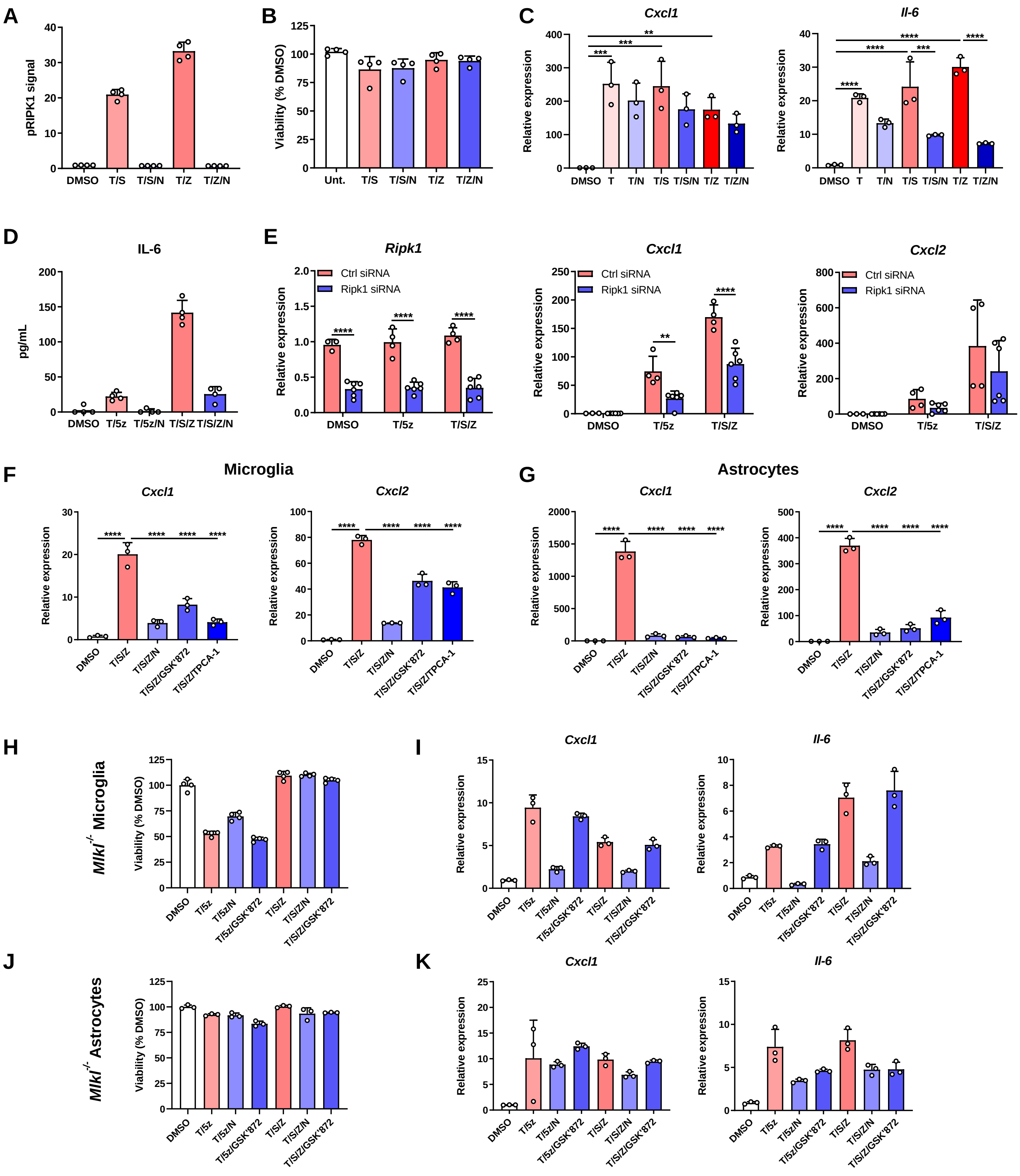
A
pRIPK1 signal
DMSO
T/S
T/S/N
T/Z
T/Z/N
B
Viability (% DMSO)
Unt.
C
Cxcl1
Il-6
Relative expression
T
T/N
D
IL-6
pg/mL
T/5z
T/5z/N
T/S/Z
T/S/Z/N
E
Ripk1
Cxcl1
Cxcl2
Ctrl siRNA
Ripk1 siRNA
F
Microglia
G
Astrocytes
T/S/Z/GSK'872
T/S/Z/TPCA-1
H
Mlkl-/- Microglia
I
T/5z/GSK'872
T/Z/N
T/Z/GSK'872
J
Mlkl-/- Astrocytes
K

Figure S4. RIPK1 kinase activation regulates pro-inflammatory signaling independently of MLKL and cell death in murine astrocytes and microglia *in vitro*, Related to **Figure 3**
(A) MSD quantification of pRIPK1 levels in astrocytes treated for 2h.
(B) Viability of astrocytes stimulated for 20h (n=4).
(C) Relative gene expression in astrocytes treated for 4h (n=3).
(D) IL-6 levels in astrocytes as determined by ELISA after 20h treatment.
(E) Relative gene expression in astrocytes treated with *Ripk1* siRNA for 72h then stimulated for 4h as indicated (n=3 for DMSO Ctrl siRNA, n=4 for T/5z and T/S/Z Ctrl siRNA, and n=6 for *Ripk1* siRNA).
(F-K) Relative gene expression in microglia **(F)** or astrocytes **(G)** treated for 4h with T/S/Z, with or without Nec-1s, RIPK3 kinase inhibitor GSK'872, or IKKα/β inhibitor TPCA-1 (n=3). Viability of *Mlkl*$^{-/-}$ microglia **(H)** and astrocytes **(J)** stimulated for 20h with T/5z or T/S/Z, with or without Nec-1s or GSK'872. Relative gene expression in *Mlkl*$^{-/-}$ microglia **(I)** or astrocytes **(K)** treated for 4h with T/5z or T/S/Z, with or without Nec-1s or GSK'872.
Error bars represent mean ± SD. Data depict technical replicates and are representative of 2 **(A and H-K)** or 3 **(D)** independent experiments. One-way ANOVA with Tukey **(C)** or Dunnett post-hoc test **(F, G)**, or two-way ANOVA with Sidak test **(E)** was performed.
** p<0.01, *** p<0.001, **** p<0.0001. T: TNF, 5z: 5z-7, S: Smac, Z: zVAD, N: Nec-1s.

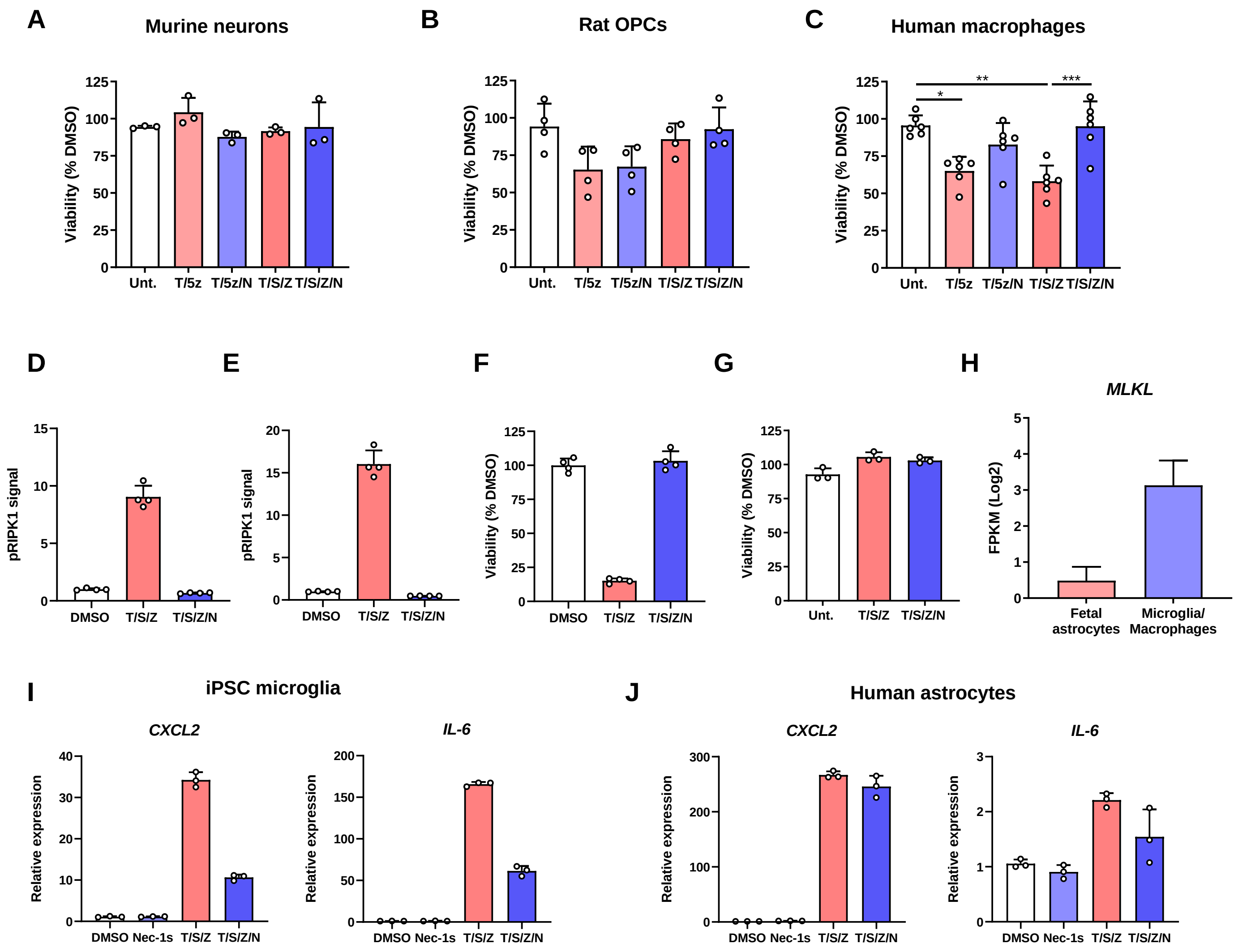

K

Comparison	↑ genes	↓ genes	Total
T/S/Z-induced genes	1132	1104	2236
RIPK1-dependent	61	100	161

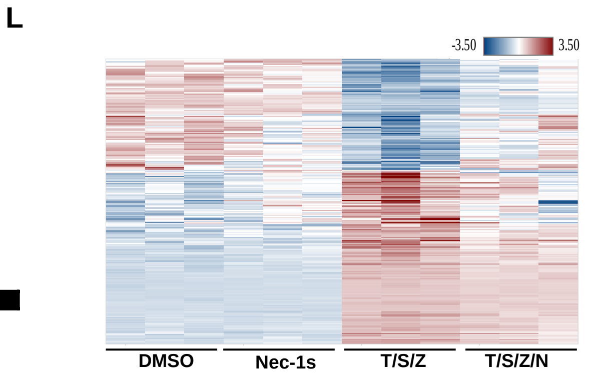

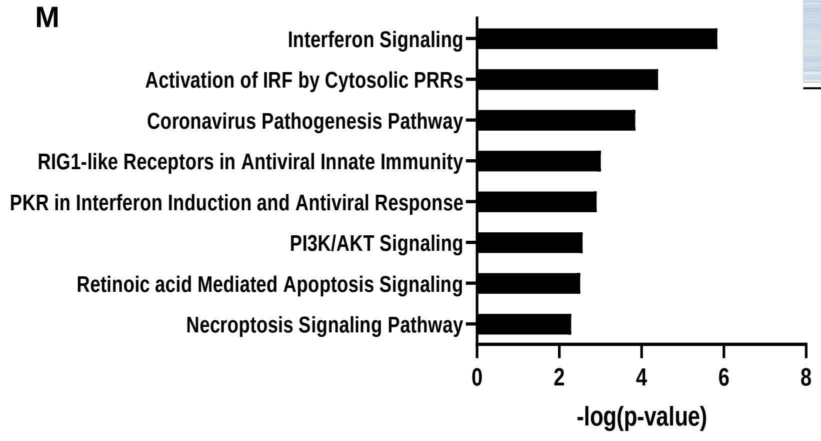

Figure S5. RIPK1 kinase activation in human cells mediates inflammatory signaling and cell death, Related to **Figures 3 and 4**

(A-C) Viability of primary murine neurons **(A)**, rat OPCs **(B)**, and human macrophages **(C)** stimulated for 20h (n=3 for neurons, n=4 for OPCs, and n=6 for macrophages).

(D, E) MSD assay quantifying pRIPK1 levels in human iPSC-derived microglia **(D)** and human fetal-derived astrocytes **(E)** treated for 2h.

(F, G) Viability of human iPSC-derived microglia **(F)** and human fetal-derived astrocytes **(G)** stimulated for 20h.

(H) *MLKL* gene expression levels in human fetal astrocytes and human microglia/macrophages from a published RNA-seq database (Zhang et al., 2016) (n=3).

(I, J) Relative gene expression in iPSC-derived microglia **(I)** or human fetal-derived astrocytes **(J)** treated for 4h.

(K) RNA-seq data in human fetal-derived astrocytes comparing upregulated and downregulated genes upon T/S/Z or T/S/Z/N (RIPK1-dependent) stimulation (cut-off $|FC|>1.3$, $p<0.05$).

(L) Hierarchical heatmap clustering of RIPK1 kinase-dependent genes in response to indicated stimuli (cut-off $|FC|>1.3$, $p<0.05$ for T/S/Z vs. DMSO, with RIPK1 kinase-dependent genes changing in opposite direction with Nec-1s addition and $p<0.05$).

(M) Top pathways predicted from IPA for RIPK1 kinase-dependent genes from **(L)**.

Error bars represent mean ± SD. Data depict technical replicates and are representative of 2 **(D-G and I-J)** independent experiments. One-way ANOVA with Tukey post-hoc test was performed in **(C)**. * $p<0.05$, ** $p<0.01$, *** $p<0.001$. T: TNF, 5z: 5z-7, S: Smac, Z: zVAD, N: Nec-1s.

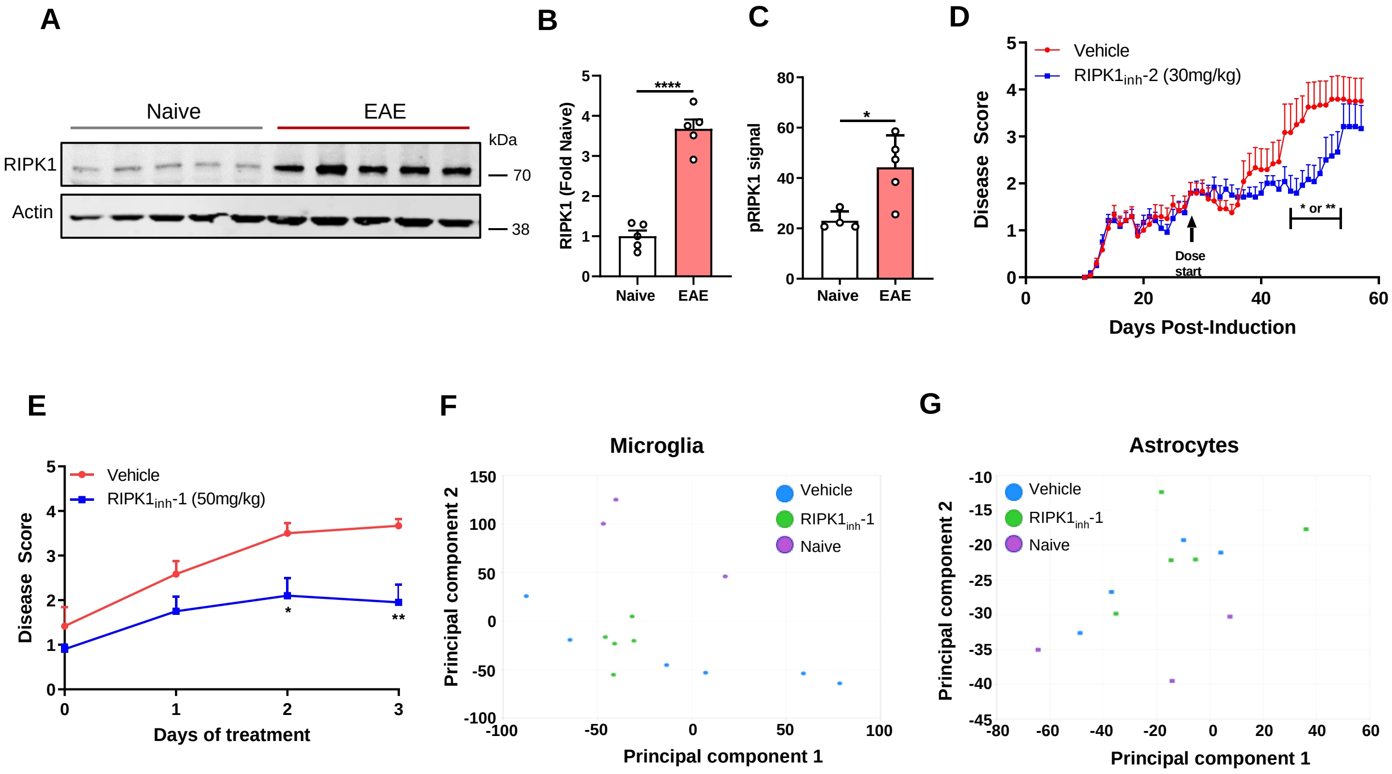

Figure S6. RIPK1 kinase inhibition attenuates disease scores in a NOD-EAE model of progressive disease and RIPK1 kinase activity regulates various signaling pathways in microglia and astrocytes during EAE**,** Related to **Figure 6**

(A) Immunoblot demonstrating RIPK1 expression in the insoluble fraction of spinal cords from untreated NOD-EAE mice (disease score of 3) compared to naïve animals (n=5 mice/group).

(B) Quantification of total RIPK1 protein levels in **(A)** normalized to naïve control.

(C) MSD quantification of pRIPK1 levels in samples from **(A)** with (n=4 naïve and n=5 EAE samples).

(D) EAE was induced in NOD mice and disease progression was monitored, demonstrating the initial relapsing/remitting and subsequent progressive stages of the MOG-induced NOD-EAE model. Mice were randomized into treatment groups 28 days after disease induction. Mean disease scores for vehicle (0.5% methylcellulose, BID) and RIPK1 kinase inhibitor groups ($RIPK1_{inh}$-2, 30mg/kg BID) were monitored daily (n=12 mice/group).

(E) EAE was induced in C57BL/6 mice and daily mean disease scores are depicted for vehicle (20% Captisol, n=6 mice) and RIPK1 kinase inhibitor groups ($RIPK1_{inh}$-1, 50mg/kg bid, n=5 mice).

(F, G) Principal component analysis of RNA-seq data in microglia **(F)** and astrocytes **(G)** isolated from spinal cords of mice in **(E)**.

Error bars represent mean ± SEM **(B-E)**. Unpaired two-tailed Student's *t* test with Welch's correction **(B and C)**, or two-way ANOVA with Dunnett **(D)** or Sidak test **(E)** was performed. * $p<0.05$, ** $p<0.01$, **** $p<0.0001$ with a significant difference between groups from days 45 to 53 in **(D)**.

Sample ID	Brain ID	Sex	Sample Type	Source	RIN	PMI (h)
1	5293	F	NAWM	UCLA	8.2	11.5
2	4823	F	NAWM	UCLA	6.4/5.3	11.8
3	5214	M	NAWM	UCLA	8	19.5
4	4621	F	NAWM	UCLA	7.1	17.6
5	4514	M	NAWM	UCLA	7.5	17.3
6	5154	M	RRMS	UCLA	7	12.6
7	5139	M	RRMS	UCLA	7.1	36.8
8	5095	M	RRMS	UCLA	9.5	8.1
9	5102	F	RRMS	UCLA	8.3	14.7
10	5123	F	RRMS	UCLA	2	24.8
11	5053	M	SPMS	UCLA	8.2	24.9
12	5056	F	SPMS	UCLA	8	20.1
13	5276	F	SPMS	UCLA	8.5	16.9
14	4959	F	SPMS	UCLA	7.5	21.1
15	4678	M	SPMS	UCLA	6.4	13.6
16	4961	F	PPMS	UCLA	7.8	9.3
17	4951	F	PPMS	UCLA	7.5	24.8
18	2485	M	PPMS	UCLA	7.5	9
19	5149	M	PPMS	UCLA	8	11
20	3816	F	PPMS	UCLA	8.7	20.7
21	4399	M	PPMS	UCLA	7.7	7.4
22	5252	M	SPMS	UCLA	9.1	10.5
23	4307	M	NAWM	UCLA	8.3	11.8
24	4611	M	NAWM	UCLA	N/A	22.3
25	4660	F	NAWM	UCLA	N/A	18.5
26	5233	F	NAWM	UCLA	N/A	20.5
27	4064	F	NAWM	UCLA	N/A	15.4
28	4135	M	NAWM	UCLA	N/A	12.6
29	5068	N/A	SPMS	UCLA	9	N/A
30	5093	N/A	SPMS	UCLA	8.7	N/A
31	4605	N/A	SPMS	UCLA	9.6	N/A
32	4732	N/A	SPMS	UCLA	7.7	N/A
33	4378	F	Prog. MS	UCLA	N/A	13.1
34	4546	M	Prog. MS	UCLA	N/A	38.5
35	4212	F	Prog. MS	UCLA	N/A	18.9
36	4218	F	Prog. MS	UCLA	N/A	15
37	4605	F	Prog. MS	UCLA	N/A	11.8
38	4629	F	Prog. MS	UCLA	N/A	15.2
39	4659	F	Prog. MS	UCLA	N/A	21.1

Sample ID	Brain ID	Sex	Sample Type	Source	RIN	PMI (h)
40	4663	M	Prog. MS	UCLA	N/A	16.1
41	4523	F	Prog. MS	UCLA	N/A	22
42	4107	F	Prog. MS	UCLA	N/A	20.6
43	4700	F	Prog. MS	UCLA	N/A	24.8
44	4467	F	Prog. MS	UCLA	N/A	22.9
45	4477	F	Prog. MS	UCLA	N/A	18.8

Table S1. Human brain samples, Related to **Figures 1 and S1**
Data table for human samples used in MS tissue analysis described in Figures 1 and S1. RIN: RNA integrity number. PMI: Post-mortem interval. Prog. MS: Progressive MS.
